# Single‐Cell RNA Sequencing and Bulk RNA Sequencing Revealed the Interplay Between Intratumoral Heterogeneity and the Tumor Microenvironment in Breast Cancer

**DOI:** 10.1002/cam4.71600

**Published:** 2026-02-03

**Authors:** Yunlong Zhao, Xiaoyu Zhang, Yingying Wang, Xiaomin Yu, Fengchun Lv, Mingyu Gong, Xiu‐An Yang

**Affiliations:** ^1^ Laboratory of Gene Engineering and Genomics, School of Basic Medical Sciences Chengde Medical University Chengde China; ^2^ Graduate School of Chengde Medical University Chengde China; ^3^ Hebei Key Laboratory of Nerve Injury and Repair Chengde Medical University Chengde China

**Keywords:** breast tumor, communication, single‐cell RNA sequencing, tumor microenvironment

## Abstract

**Objective:**

The study is to investigate differential signaling pathways within the tumor microenvironment across molecular subtypes of breast cancer (BC).

**Methods:**

Single‐cell RNA (scRNA‐seq) sequencing data of BC samples were obtained from the Gene Expression Omnibus database. Cell types were identified using the SingleR package, in conjunction with the analysis of marker genes. Subsequently, Monocle was used for pseudotime analysis of epithelial cells, fibroblasts, and macrophages to characterize their differentiation states. CellChat was employed to study the ligand‐receptor interactions among various cell types across different BC molecular subtypes. In addition, we used common bulk RNA sequencing data from The Cancer Genome Atlas to investigate the correlation between key signaling pathway factors identified by scRNA‐seq and clinical outcomes.

**Results:**

Inference of copy number variation analysis using T cells revealed significantly elevated copy number variation scores in epithelial cells and fibroblasts. In the communication between epithelial cells and fibroblasts, the ANGPTL pathway is critical in estrogen receptor‐positive breast cancer (ER+BC), while the PTN pathway plays a key role in both ER+BC and human epidermal growth factor receptor 2‐positive breast cancer (HER2+BC), and the GAS pathway is associated with poor prognosis in triple‐negative breast cancer (TNBC). In the interaction between fibroblasts and macrophages, the macrophage subpopulation supporting tumor angiogenesis exhibits significant activity in ER+BC, with the associated SPP1 and GRN pathways strongly influencing tumor progression. The SEMA3 pathway mainly acts through dividing tumor‐associated fibroblast clusters across all BC subtypes. When exploring the role of lymphocytes, the PTN pathway also plays a role in HER2+BC, while in TNBC, CXCL and CD70 pathways are significantly involved in immune response modulation.

**Conclusion:**

Our comprehensive analysis of cell–cell communication networks among epithelial cells, fibroblasts, macrophages, and lymphocytes across BC subtypes focuses on ligand‐receptor interactions. This study revealed that certain molecules within these networks exhibit significant prognostic value and therapeutic promise.

## Graphical abstract

The figure shows single‐cell datasets of breast cancer collected from the GEO database, including estrogen receptor‐positive breast cancer, HER2‐positive breast cancer, and triple‐negative breast cancer. These datasets are used to analyze differences among breast cancer subtypes by studying the interactions among epithelial cells, fibroblasts, macrophages, and lymphocytes.

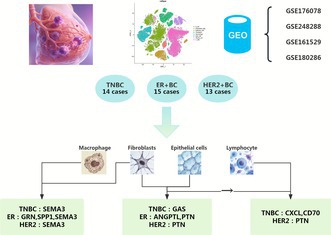

## 1 Introduction

Breast cancer (BC) is the most frequently diagnosed cancer in women worldwide and it is the leading cause of cancer‐related deaths [1]. Globally, 2.3 million new cases and 670,000 deaths from BC in females occurred in 2022 [2], profoundly impacting both individual lives and healthcare systems [3]. Despite advances in surgery, chemotherapy, and immunotherapy, clinical outcomes remain suboptimal for many BC patients, even in early‐stage disease [4]. These findings demonstrate the urgent need for both improved treatment options and validated prognostic biomarkers.

BC is a highly heterogeneous disease that is clinically classified according to the expression status of the estrogen receptor (ER), progesterone receptor (PR), and human epidermal growth factor receptor 2 (HER2). Based on these markers, it is categorized into three major clinical groups with distinct prognostic and therapeutic implications: estrogen receptor‐positive breast cancer (ER+BC), human epidermal growth factor receptor 2‐positive breast cancer (HER2+BC), and triple‐negative breast cancer (TNBC) [5]. Among these subpopulations, estrogen receptor‐positive and HER2‐negative breast cancer (ER+/HER2‐BC) makes up approximately 60%–70% of all BC [6, 7]. TNBC represents 10%–20% of all BC cases [8], which has the worst prognosis among BC subtypes [9].

To enable effective tumor control and management, a thorough understanding of the molecular pathways underlying carcinogenesis and tumor development is essential. The epithelial‐mesenchymal transition (EMT) converts epithelial cells into mesenchymal cells and facilitates cancer stem cell formation. Additionally, EMT enhances the tumor cells (TCs) invasion, migration, and metastasis. TNBC and ER+BC both show significantly increased populations of cells carrying EMT genetic markers [10]. The vast majority (> 95%) of invasive breast carcinomas are adenocarcinomas that originate from the epithelial cells of the terminal duct‐lobular unit [11]. Therefore, research on epithelial cells is equally vital for developing therapies to block BC invasion and metastasis, reducing patient mortality, and uncovering new molecular targets for treatment.

The tumor microenvironment (TME) is a biologically complex and dynamic system composed of multiple interacting cell types, specifically cancer cells, stromal cells, immune cells, and vascular endothelial cells [12]. Tumors are ecosystems in which cancer cells engage in active crosstalk with cells of the TME, and it is through these interactions that tumors progress and metastasize. Major players in the TME of many carcinomas are cancer‐associated fibroblasts (CAFs). CAFs support diverse protumorigenic functions, including extracellular matrix (ECM) remodeling, immune evasion, angiogenesis, and invasion [13]. Studies confirm that cancer cells actively appropriate fibroblast activation processes and utilize wound repair mechanisms to facilitate tumor growth during cancer progression [14], highlighting CAFs' critical role in supporting tumor development. Meanwhile, CAFs, as a major constituent of the breast TME, are a heterogeneous population of cells with an emerging role in modulating antitumor immunity and influencing responses to treatment [15, 16, 17]. Tumor‐infiltrating immune cells (TIICs), particularly tumor‐associated macrophages (TAMs), constitute another essential element of the TME that critically regulates tumor growth, metastatic spread, and treatment responses [18]. Yavuz et al. demonstrated that CAFs isolated from human invasive BC could facilitate the differentiation of monocytes into M2‐like pro‐tumoral macrophages. This effect contrasts with that of normal breast‐derived fibroblasts [19]. CAFs can also promote skin tumor development by maintaining monocyte chemotactic protein‐1 (MCP‐1) mediated macrophage infiltration and chronic inflammation [20]. Furthermore, the interplay between CAFs and TAMs seems to be very complex, as these two groups of cells are also able to alter each other's functions [21]. Current therapeutic paradigms in oncology—including both immunotherapeutic and chemotherapeutic approaches—critically overlook the essential pathophysiological roles of CAFs in mediating treatment resistance and tumor progression [22]. Consequently, comprehensive investigation of the dynamic interplay between CAFs and TAMs within the TME is imperative for developing transformative therapeutic strategies.

In TIICs, tumor‐infiltrating lymphocytes (TILs) are equally important. Evidence shows that TILs present in pre‐treatment BC can predict treatment response and improve prognosis [23, 24]. Similarly, a retrospective study of 53 mastectomy samples found that the infiltration of B cells and T cells increased in benign ductal hyperplasia, further increased in ductal carcinoma in situ (DCIS), and showed the greatest increase in invasive BC [25]. Importantly, compared to TNBC and HER2+BC, the levels of TILs are lower in hormone receptor‐positive and HER2‐negative (HR+/HER2−) tumors [26]. Given the complex mechanisms of TILs, it is critical to explore their role in different BC subtypes in depth.

Single‐cell RNA sequencing (scRNA‐seq) has demonstrated its transformative power in deconvoluting the heterogeneity within tumor ecosystems by deciphering cell type specific transcriptomic profiles across both malignant and microenvironmental compartments, and systematically charting the bidirectional crosstalk between neoplastic cell populations and stromal elements [27, 28, 29]. This powerful technological advancement is invaluable for elucidating intercellular communication networks and uncovering new clinical intervention strategies. In oncology, integrating scRNA‐seq with prior knowledge allows for comprehensive mapping of cellular interactions and precise identification of cancerous cell populations, offering unprecedented resolution to study tumor pathobiology and disease origins [30]. Despite its applications in the TME of BC research, broader application of single‐cell transcriptomics in BC studies remains limited. In this study, we conducted a multi‐omics joint analysis of scRNA‐seq data and bulk RNA sequencing (bulk RNA‐seq) of BC. We aimed to gain insights into the tissue‐specific characteristics of BC in response to different hormone levels at the single‐cell level, and identify key genes and their correlation with clinical outcomes. These findings provided valuable insights into the etiology and progression of BC, which are greatly helpful for improving treatment methods.

## 2 Materials and Methods

### 2.1 Data Source

In this study, we analyzed 42 primary BC samples from publicly available datasets in the Gene Expression Omnibus database (GEO). These included all samples from GSE176078 (11 ER+, 5 HER2+, 10 TNBC) [31] and GSE248288 (4 ER+), as well as selected samples from GSE161529 (6 HER2+, 4 TNBC) and GSE180286 (2 HER2+). Altogether, the dataset comprised 15 ER+BC, 14 TNBC, and 13 HER2+BC cases. No ethical approval was required, as only publicly available data were used. Additionally, transcriptome data of BC patients were collected from The Cancer Genome Atlas (TCGA), which included 486 ER+BC cases, 30 HER2+BC cases, and 123 TNBC cases. Patient details are provided in Table S1.

### 2.2 Single Cell Data Processing

Following data import into R (v4.2.0), scRNA‐seq analysis was performed using Seurat (v4.3.0) according to established computational workflows. To ensure analytical rigor, we implemented stringent quality control measures with the following thresholds: Cells expressing fewer than 100 or more than 50,000 genes were excluded. High‐quality cells were defined as those with unique molecular identifier (UMI) counts greater than 500, mitochondrial UMI ratio less than 10%, and red blood cell gene ratio less than 1%. Technical doublets were computationally identified using DoubletFinder and subsequently removed from the analysis.

Gene expression data were normalized and scaled using Seurat's built‐in functions: NormalizeData (log‐normalization with a scale factor of 10,000) and ScaleData (linear scaling with mitochondrial percentage regression). The top 3000 most highly variable genes were identified using the FindVariableFeatures function as input for principal component analysis (PCA). PCA was performed using the RunPCA function, followed by batch effect correction using the RunHarmony function from the Harmony package. Cellular clustering was performed using the FindNeighbors and FindClusters functions with 40 principal components (PCs), followed by dimensionality reduction through the t‐distributed Stochastic Neighbor Embedding (t‐SNE) method. Following dimensionality reduction, cell type annotation was performed through an integrated approach combining reference‐based classification using SingleR with marker gene expression analysis.

### 2.3 Gene Set Functional Analysis

Comprehensive gene set enrichment and pathway activity analysis was performed through an integrated computational workflow. First, the MSigDB Hallmark 2020 and WikiPathway 2021 Human gene sets were acquired from the Enrichr knowledgebase (https://maayanlab.cloud/Enrichr/). Using the Enrichr R package, we conducted gene set enrichment analysis focused on differentially expressed marker genes from epithelial and fibroblast subpopulations. Pathway selection was conducted using stringent dual criteria, which combined statistical significance (a false discovery rate (FDR) corrected *p*‐value of < 0.05 was used as the threshold) with biological relevance, specifically prioritizing pathways implicated in epithelial–mesenchymal interactions and the TME modulation. For single‐cell resolution pathway activity assessment, we implemented the AUCell computational framework in three stages: input the raw matrix constructed by Seurat to perform cell‐specific gene ranking (AUCell_buildRankings), pathway enrichment scores calculation using area under the curve metrics (AUCell_calcAUC), and generation of clustered heatmaps to visualize pathway activation patterns across cell subpopulations. Consequently, this multi‐modal approach enabled systematic identification of biologically relevant pathways and their activity patterns at single‐cell resolution.

### 2.4 InferCNV Analysis

Inference of copy number variation (inferCNV) analysis was performed using the inferCNV R package to systematically inspect genomic alterations between epithelial cells and fibroblasts. The workflow incorporated three essential input files: raw gene expression counts, cell type annotations (specifying epithelial, fibroblast, and reference T cell populations), and gene chromosomal coordinates. Analysis parameters included T cells as diploid controls, a 2.5 standard deviation threshold for noise reduction, and default settings for remaining parameters. Significant Copy number variations (CNVs) were identified as genomic regions exhibiting > 3 SD deviations from the reference, with recurrent alterations mapped to specific chromosomal loci to distinguish epithelial‐specific from fibroblast‐specific variants.

### 2.5 Cell–Cell Communication Analysis

Using the CellChat package in R with reference to the CellChatDB database, we systematically analyzed secretory signaling‐mediated cell–cell communication networks across all identified cell populations in our dataset. Comprehensive cell–cell communication analysis was performed using CellChat through a multi‐stage computational workflow. First, interaction probabilities were calculated using the computeCommunProb function, followed by stringent filtering (min.cells = 10) to eliminate spurious connections. Pathway‐level communication strength was then quantified via computeCommunProbPathway, with integrated network visualization through aggregateNet. Key biological research systematically characterized the signaling between epithelial cells and fibroblasts, as well as between fibroblasts and macrophages, in various subtypes of BC. Additionally, it analyzed the communication networks of lymphocytes with epithelial cells and fibroblasts in HER2+BC and TNBC, respectively.

### 2.6 Pseudotime Trajectory Analysis

Monocle was employed for single‐cell pseudotemporal ordering to delineate differentiation pathways among epithelial, fibroblast, and macrophage subpopulations. Utilizing the DDRTree algorithm within the reduce Dimension function, a minimum spanning tree was then built to outline cell differentiation paths. This method guarantees connectivity among cells while reducing the cumulative edge weight. Finally, the derived trajectory was depicted using the plot_cell_trajectory function.

### 2.7 Single‐Cell Regulatory Network Inference and Clustering (SCENIC)

To delve deeper into the mechanisms underlying the heterogeneity among various cell clusters and to examine the expression patterns of differentially expressed genes, we utilized single‐cell regulatory network inference and clustering to construct a transcription factor regulatory network (SCENIC, version 1.1.0.1). In essence, SCENIC identifies co‐expression modules between transcription factors (TFs) and potential target genes, as well as calculates regulatory activity scores (RAS) for individual cells. The regulatory specificity score (RSS) is calculated to forecast the precise relationship between regulatory factors and specific cell types. The connectivity specificity index (CSI) is employed to indicate the interplay between different regulatory rules. Regulatory genes with a higher CSI are likely to co‐regulate downstream genes and collectively contribute to cellular functions. The study used SCENIC to identify TFs with higher activity in epithelial cell subpopulations across different BC subtypes and further elucidated the communication between epithelial cells and fibroblasts through these TFs.

### 2.8 Analysis of BC Bulk RNA‐Seq

This study utilizes transcriptome data from various BC subtypes downloaded from the TCGA database. These data are integrated with key factors from important pathways identified through CellChat analysis, as well as clinical factors, including age, M‐Stage, N‐Stage, T‐Stage, Pathological Stage, and survival, to assess the correlation between gene expression and clinical outcomes. Since only a single statistical test is performed, a *p*‐value < 0.05 is used to indicate statistical significance.

## 3 Results

### 3.1 Cell Types in BC

**FIGURE 1:**
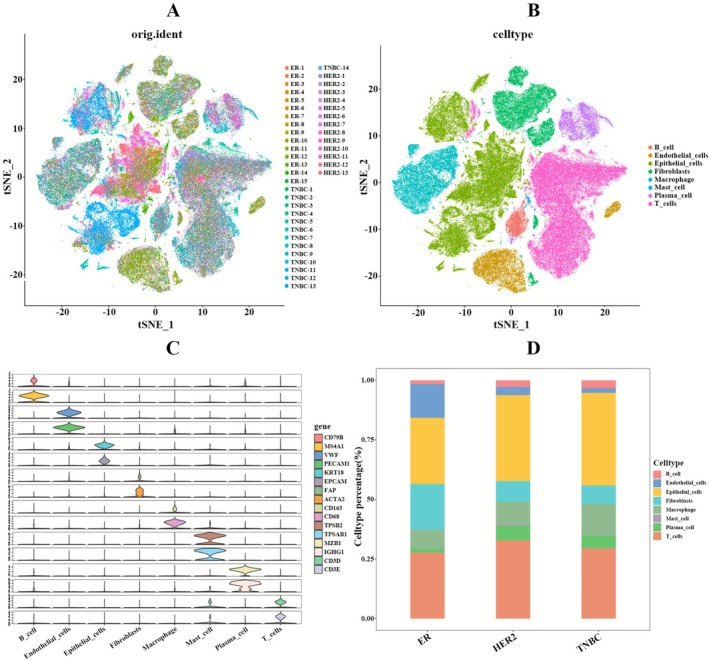
scRNA‐seq reveals distinct cellular heterogeneity and tumor heterogeneity across different BC subtypes. (A) t‐SNE plot displays the effects of different samples after batch processing. (B) t‐SNE plot reveals the distribution of eight cell types: B cells, endothelial cells, epithelial cells, fibroblasts, macrophages, mast cells, plasma cells, and T cells. (C) Violin plot shows the marker genes for cell types. (D) Bar plot of cell types reveals the differences in cell composition among BC subtypes.

To comprehensively characterize the cellular heterogeneity of breast tumor tissue, the study conducted scRNA‐seq on 42 primary BC specimens. Following rigorous quality control, including doublet detection and removal, a total of 157,848 high‐quality cells were retained for subsequent analysis. The processed data underwent normalization, variance stabilization, dimensionality reduction, and graph clustering, with technical artifacts mitigated using Harmony integration. This computational pipeline effectively harmonized the dataset across all samples (Figure 1A). For precise cell type identification, a dual validation approach was implemented, combining computational annotation with SingleR and manual verification using well‐established lineage‐specific markers, collectively identifying eight distinct cell populations (Figure 1B): B_cell (CD79B, MS4A1), Endothelial_cells (PECAM1, VWF), Epithelial_cells (EPCAM, KRT18), Fibroblasts (FAP, ACTA2), Macrophage (CD163, CD68), Mast_cell (TPSB2, TPSAB1), Plasma_cell (MZB1, IGHG1), T_cells (CD3D, CD3E) (Figure 1C).

The cellular composition analysis (Figure 1D) demonstrated distinct patterns among the three BC subtypes (ER+BC, HER2+BC, and TNBC). Epithelial cells, representing the primary malignant cell population, constituted the predominant fraction in all subtypes. Notably, ER+BC specimens exhibited elevated proportions of both endothelial cells and fibroblasts compared to other subtypes. This observation corroborates existing literature [32] that identifies fibroblasts as crucial mediators of immune responses within ER+BC tumors, potentially explaining this subtype's unique pathological characteristics. While substantial immune cell populations were detected in all three subtypes, the absence of spatial context in scRNA‐seq data limits functional interpretation. Notably, only in TNBC and HER2+BC are high levels of TILs consistently linked to clear clinical significance [26]. These findings suggest that subtype specific variations in cellular composition may underlie their differential clinical manifestations and treatment responses.

### 3.2 InferCNV and Functional Enrichment Analysis of Epithelial Cells

**FIGURE 2:**
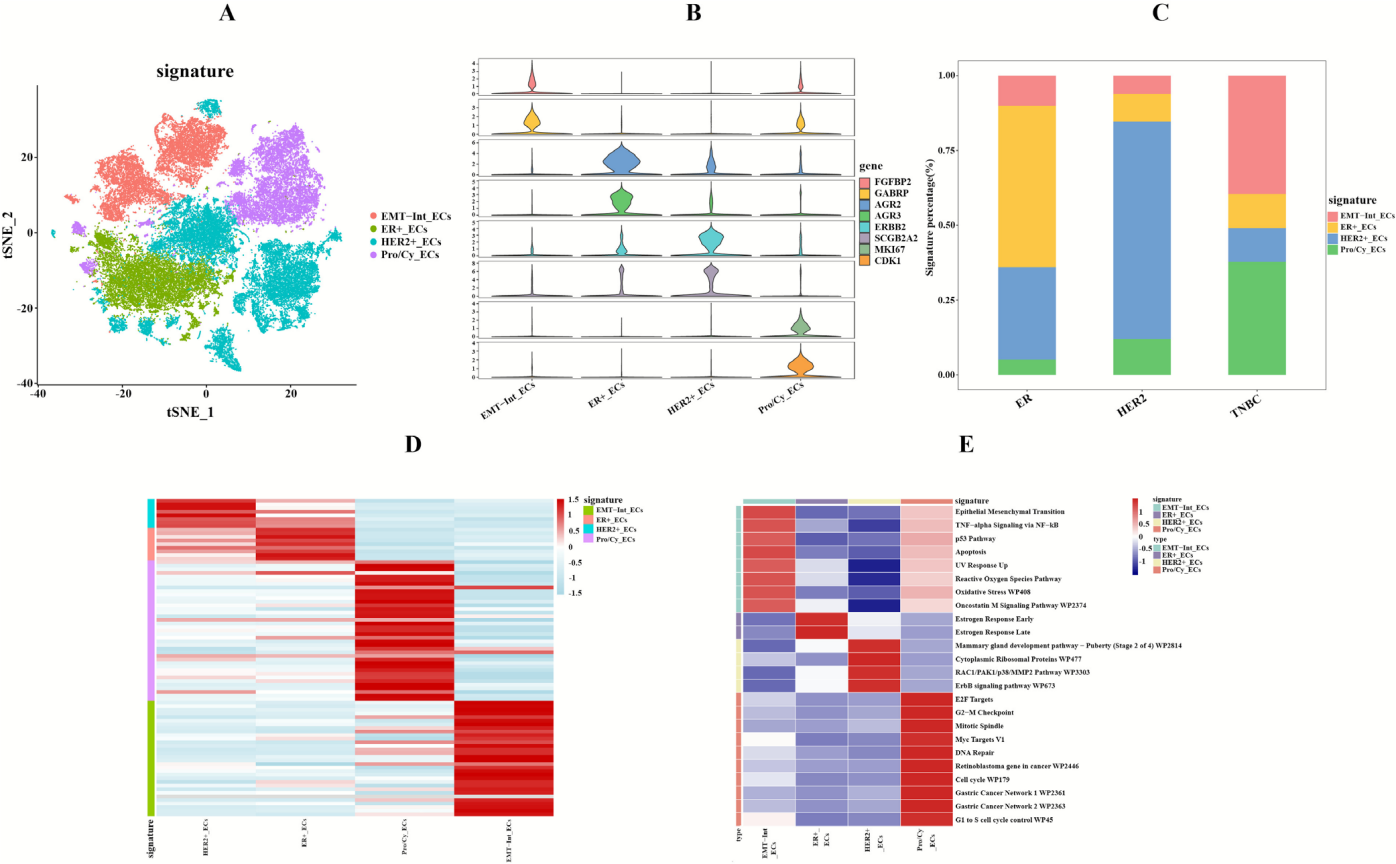
Subtyping of epithelial cells and enrichment analysis of signaling pathways. (A) t‐SNE plot displays the distribution of epithelial cell subpopulations, labeled as HER2+_ECs, ER+_ECs, Pro/Cy_ECs, and EMT‐Int_ECs. (B) Violin plot shows the marker genes for epithelial cell subpopulations. (C) Bar plot showing the proportional distribution of epithelial cell subpopulations across different BC subtypes. (D) Heatmap displays expression patterns of differentially expressed genes across subpopulations. (E) Heatmap depicts pathway enrichment patterns among epithelial cell subpopulations.

Breast malignancies predominantly originate from mammary epithelial cells, highlighting the critical role of epithelial cell mutations in BC pathogenesis. Following stringent quality control procedures, 54,674 high‐quality cells were selected for subsequent analysis. The epithelial cell subset then underwent standardized computational processing, including batch effect correction, dimensionality reduction through principal component analysis, and unsupervised clustering. Using the FindAllMarkers function (logFC > 1), and integrating these results with previous studies, we identified four distinct epithelial subpopulations (Figure 2A): HER2+_ECs (ERBB2, SCGB2A2) was predominated in HER2+BC cases; ER+_ECs (AGR2, AGR3) was dominated in ER+BC; Pro/Cy_ECs (MKI67, CDK1) and EMT‐Int_ECs (FGFBP2, GABRP) were markedly enriched in TNBC (Figure 2B,C) [33]. The subsequent heatmap, generated by integrating the expression profiles of genes highly expressed in various cancer subtypes identified in previous studies, established the basis for the classification (Figure 2D). These epithelial subpopulation distributions strongly correlate with known molecular subtypes, therapeutic responses, and clinical outcomes, suggesting they represent biologically distinct entities that may serve as diagnostic classifiers, predictive biomarkers, and potential therapeutic targets for precision oncology approaches.

InferCNV analysis using T cells as diploid controls (Figure S1A) revealed significant CNV heterogeneity among epithelial subpopulations [34] (*p*‐value < 0.05, Figure S1B), with ER+_ECs, Pro/Cy_ECs and EMT‐Int_ECs showing markedly elevated CNV scores versus near‐diploid HER2+_ECs, suggesting progressive genomic instability during malignant evolution. These patterns correlated with advanced disease stage and reduced survival [35].

Pathway enrichment analysis (enrichR) of epithelial subpopulations revealed distinct oncogenic programs (Figure 2E; Table S2). Functional enrichment analysis identified HER2+_ECs as predominantly associated with HER2, showing statistically significant involvement in Mammary gland development patPuberty (Stage 2 of 4) WP2814, Cytoplasmic Ribosomal Proteins WP477, RAC1/PAK1/p38/MMP2 Pathway WP3303, and ErbB receptor‐mediated proliferation WP673. These findings indicated that HER2+_ECs promote tumor progression centered around HER2. Pathway analysis demonstrated ER+_ECs's significant enrichment in Estrogen Response pathways (early/late response) highlighting its important role in hormonal signaling within the TME. Transcriptomic profiling identified co‐enrichment of Pro/Cy_ECs and EMT‐Int_ECs in tumorigenic pathways (EMT, TNF−alpha Signaling via NF−kB, p53 Pathway, Apoptosis, UV Response Up), while Pro/Cy_ECs uniquely demonstrated cell cycle activation (E2F Targets, G2−M Checkpoint, Myc Targets V1), a proliferative signature that correlated with its elevated genomic instability, suggesting cell cycle dysregulation as a potential driver of CNV accumulation in this aggressive subpopulation. Based on their significant enrichment in high risk oncogenic pathways (EMT, cell cycle dysregulation) and elevated genomic instability (showed higher genomic instability via CNV analysis), the Pro/Cy_ECs and EMT‐Int_ECs subpopulations were prioritized for subsequent mechanistic investigations.

### 3.3 The Developmental Trajectory and Sequence of Epithelial Cell Subsets

**FIGURE 3:**
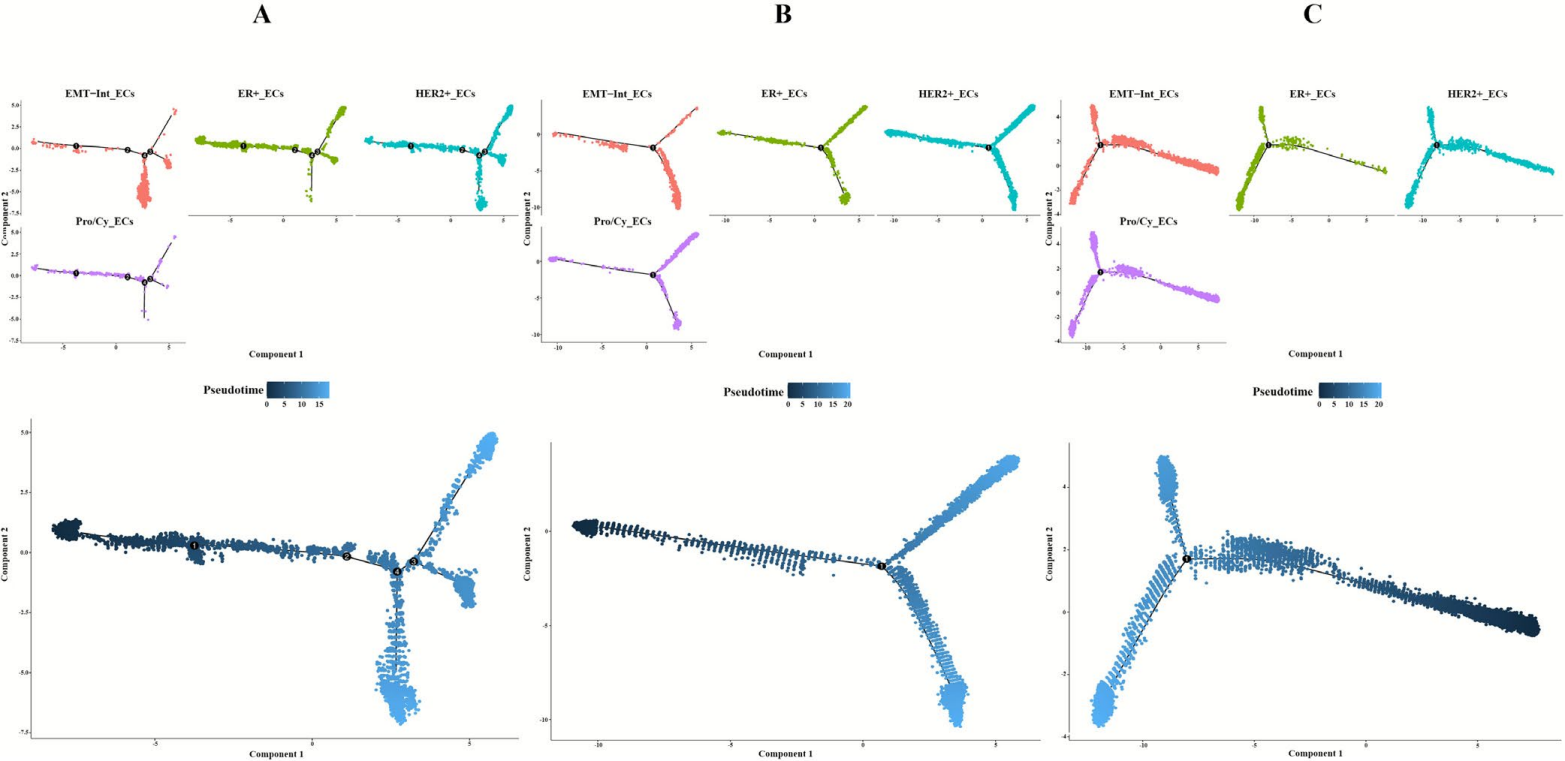
Pseudotime analysis of epithelial cell subpopulations. (A) Developmental trajectory and differentiation states of epithelial cell subpopulations in ER+BC. (B) Developmental trajectory and differentiation states of epithelial cell subpopulations in HER2+BC. (C) Developmental trajectory and differentiation states of epithelial cell subpopulations in TNBC.

To investigate the evolutionary dynamics of mammary epithelial cell lineages, pseudotemporal trajectories of epithelial subsets were computationally reconstructed across BC subtypes using Monocle. This approach enabled systematic mapping of cellular progression from non‐malignant states through malignant transformation to metastatic phenotypes. Subtype analysis revealed distinct differentiation patterns in ER+BC (Figure 3A), HER2+_ECs and ER+_ECs spanned all differentiation stages while Pro/Cy_ECs localized to intermediate stages and EMT‐Int_ECs resided in late stages. Within HER2+BC (Figure 3B), most epithelial subsets occupied late differentiation phases whereas HER2+_ECs alone exhibited substantial representation in early to mid stages. It is noteworthy that in TNBC (Figure 3C), HER2+_ECs persisted throughout differentiation while Pro/Cy_ECs and EMT‐Int_ECs populated early stages and ER+_ECs was restricted to late stages.

Our analysis showed that ER+_ECs are found exclusively in the early differentiation stage in ER+BC while HER2+_ECs have a large number of cells in the early differentiation stage predominantly in HER2+BC. Similarly, in TNBC, both Pro/Cy_ECs and EMT‐Int_ECs are in the early differentiation stage. These findings indicate that, across different BC subtypes, the predominant epithelial cell subpopulations tend to occupy the early differentiation stage in the developmental trajectory specific to each subtype.

### 3.4 InferCNV and Functional Enrichment Analysis of Fibroblasts

**FIGURE 4:**
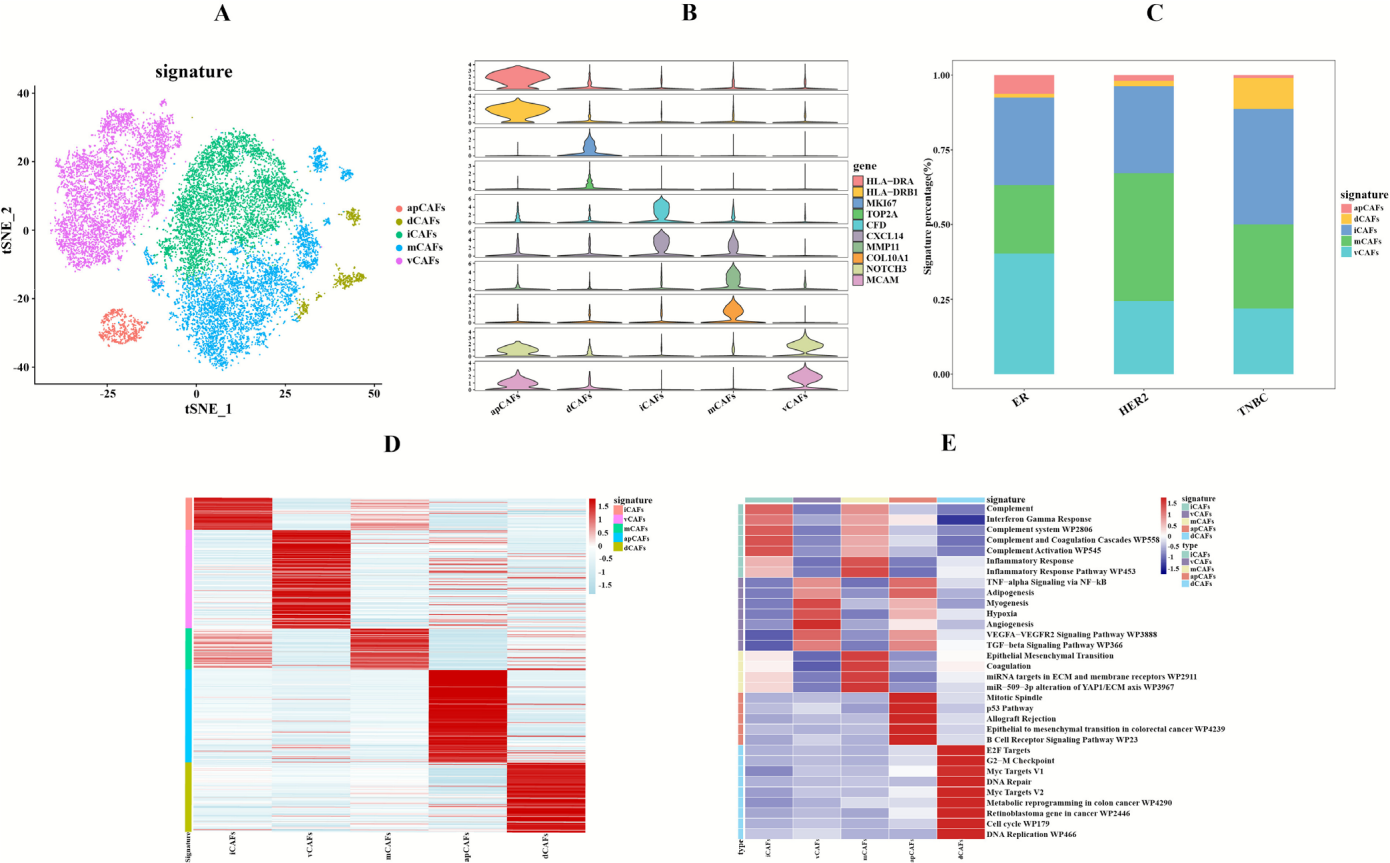
Subtyping of fibroblasts and enrichment analysis of signaling pathways. (A) t‐SNE plot displays the distribution of fibroblast subpopulations, labeled as iCAFs, vCAFs, mCAFs, apCAFs, and dCAFs. (B) Violin plot shows the marker genes for fibroblast subpopulations. (C) Bar plot shows the proportional distribution of fibroblast subpopulations across different BC subtypes. (D) Heatmap displays expression patterns of differentially expressed genes across subpopulations. (E) Heatmap depicts pathway enrichment patterns among fibroblast subpopulations.

Research has demonstrated that CAFs interact with tumor cells through two distinct mechanisms: direct cell‐to‐cell contact and paracrine secretion of signaling molecules. These interactions enable CAFs to release a repertoire of cytokines and growth factors that collectively orchestrate tumorigenesis and metastatic progression [36, 37]. Following stringent quality control of all extracted fibroblasts (*n* = 18,042), the dataset was subjected to sequential computational processing including batch‐effect correction using batch effect correction, dimensionality reduction through principal component analysis, and unsupervised clustering. Differential gene expression analysis was then performed using the FindAllMarkers function. The results were compared with those from previous studies, which identified five distinct fibroblast subpopulations (logFC > 1) (Figure 4A): iCAFs (CFD, CXCL14), vCAFs (NOTCH3, MCAM), mCAFs (COL10A1, MMP11), apCAFs (HLA−DRA, HLA−DRB1), dCAFs (MKI67, TOP2A) [38] (Figure 4B). Among apCAFs and vCAFs, these cells are mainly present in ER+BC. In contrast, mCAFs are more prevalent in HER2+BC, and dCAFs are significantly enriched in TNBC relative to other molecular subtypes (Figure 4C). Furthermore, subsequent heatmap analysis confirmed their distinct transcriptional profiles (Figure 4D).

Genomic characterization of fibroblast subpopulations was performed through InferCNV analysis to evaluate copy number variation (CNV) profiles (Figure S1C) [34]. Quantitative assessment revealed statistically significant inter‐cluster CNV heterogeneity (*p*‐value < 0.05, Figure S1D), with dCAFs exhibiting the most substantial genomic instability. The pan‐cluster distribution of CNVs supports the hypothesis of CAFs transdifferentiation potentially mediated through genomic instability pathways.

EnrichR‐based pathway profiling (Figure 4E; Table S3) revealed distinct functional specialization among CAFs subpopulations: iCAFs show significant enrichment in interferon response (IFN‐γ), complement system‐related pathways, and inflammatory responses. vCAFs are enriched in pathways related to angiogenesis, including the Angiogenesis pathway, VEGFA‐VEGFR2 Signaling Pathway WP3888, and TGF‐beta Signaling Pathway WP366. mCAFs demonstrate enrichment in matrix‐related pathways, such as miRNA targets in ECM and membrane receptors WP2911, as well as miR‐509‐3p alteration of the YAP1/ECM axis WP3967. apCAFs are enriched in pathways including Mitotic Spindle and Allograft Rejection. dCAFs exhibit the highest CNV levels and uniquely enrich in cell‐cycle pathways (specifically E2F targets, G2‐M checkpoint, and Myc targets V1), suggesting they play a dominant role in proliferation within the TNBC microenvironment.

### 3.5 The Changes in CAFs Subpopulations During Disease Progression

CAFs interact with multiple cell populations in the TME to promote tumorigenesis and progression. To characterize CAFs' developmental dynamics and differentiation states across BC subtypes, pseudotemporal trajectory analysis was performed using Monocle.

**FIGURE 5:**
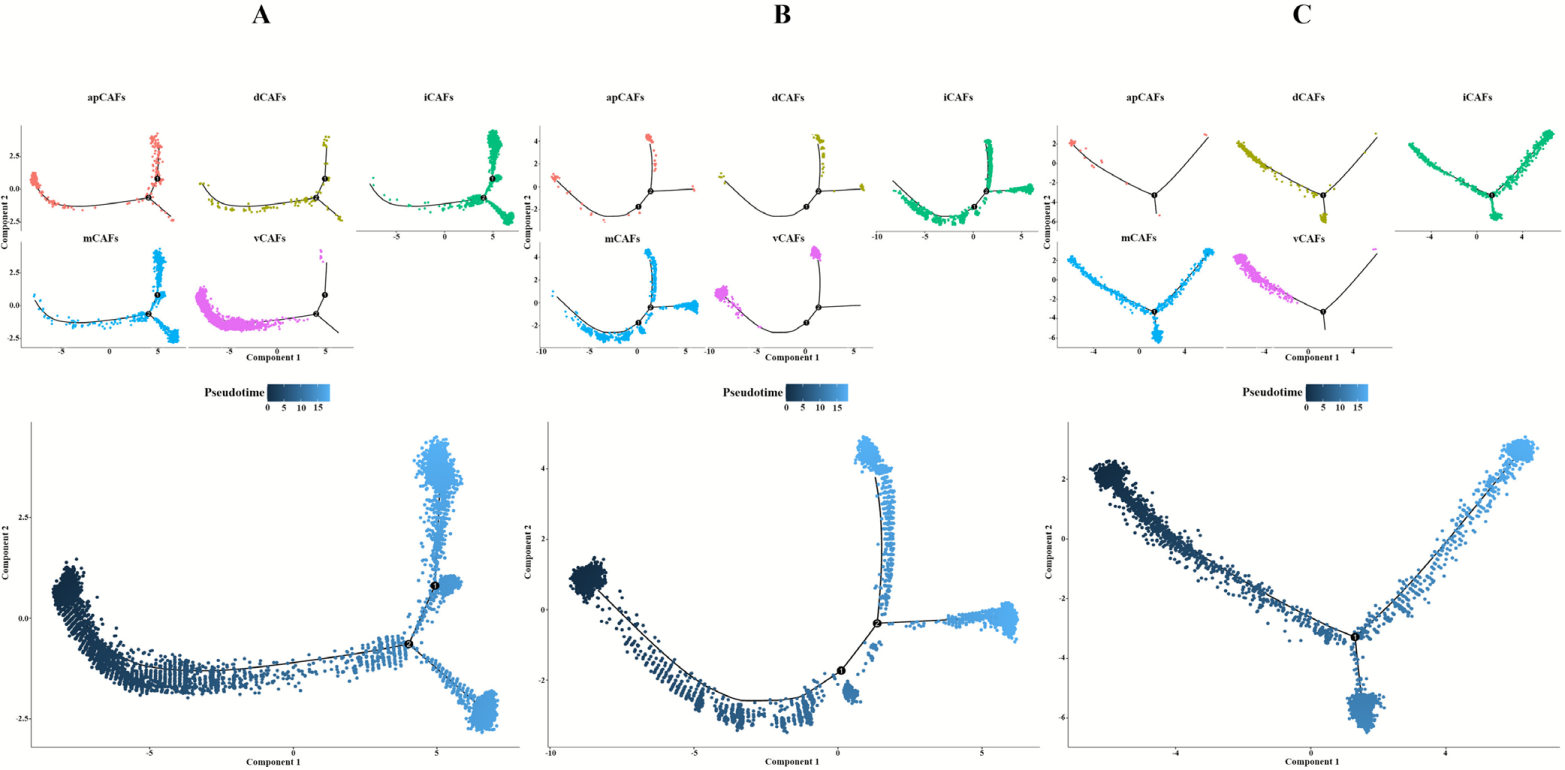
Pseudotime analysis of fibroblast subpopulations. (A) Developmental trajectory and differentiation states of fibroblast subpopulations in ER+BC. (B) Developmental trajectory and differentiation states of fibroblast subpopulations in HER2+BC. (C) Developmental trajectory and differentiation states of fibroblast subpopulations in TNBC.

Our analysis revealed distinct temporal differentiation patterns among fibroblast subpopulations across BC subtypes. In ER+BC, subpopulations apCAFs and vCAFs were predominantly localized to early differentiation while iCAFs and mCAFs were associated with terminal differentiation, with dCAFs exhibiting pan‐differentiation presence throughout all stages (Figure 5A). HER2+BC subtype showed exclusive early differentiation enrichment of vCAFs, mid‐to‐late stage distribution of iCAFs and mCAFs, and terminal restriction of apCAFs and dCAFs (Figure 5B). Notably, in TNBC, apCAFs, dCAFs and vCAFs collectively occupied progenitor‐like early stages, mCAFs displayed intermediate differentiation, and iCAFs were uniquely committed to terminal differentiation, demonstrating subtype‐specific fibroblast dynamics (Figure 5C).

Comprehensive differentiation analysis shows dCAFs, with the highest CNV burden, are present only in the early stage of differentiation and are most abundant in TNBC. The apCAFs and vCAFs, CAF subtypes that have a higher proportion in ER+BC, are also in the early stage of differentiation. Additionally, vCAFs, which have a relatively high proportion in HER2+BC, are in the early stage of differentiation. The similarity in differentiation behavior between fibroblast subtypes and epithelial cell subtypes highlights the necessity of conducting functional studies on both to establish causal mechanisms.

### 3.6 Signaling Communication Between Epithelial Cells and CAFs

Substantial evidence confirms that the interactions of fibroblast and tumor cells through both juxtacrine contact and paracrine signaling critically promote tumor progression and metastatic competence [39]. To systematically decode intercellular communication networks across BC subtypes, CellChat analysis was performed by designating epithelial subpopulations as ligand sources and CAF subpopulations as receptor targets, with stringent interaction probability thresholds applied. This computational approach enabled comprehensive mapping of putative ligand‐receptor interactions while accounting for subtype‐specific signaling patterns.

**FIGURE 6:**
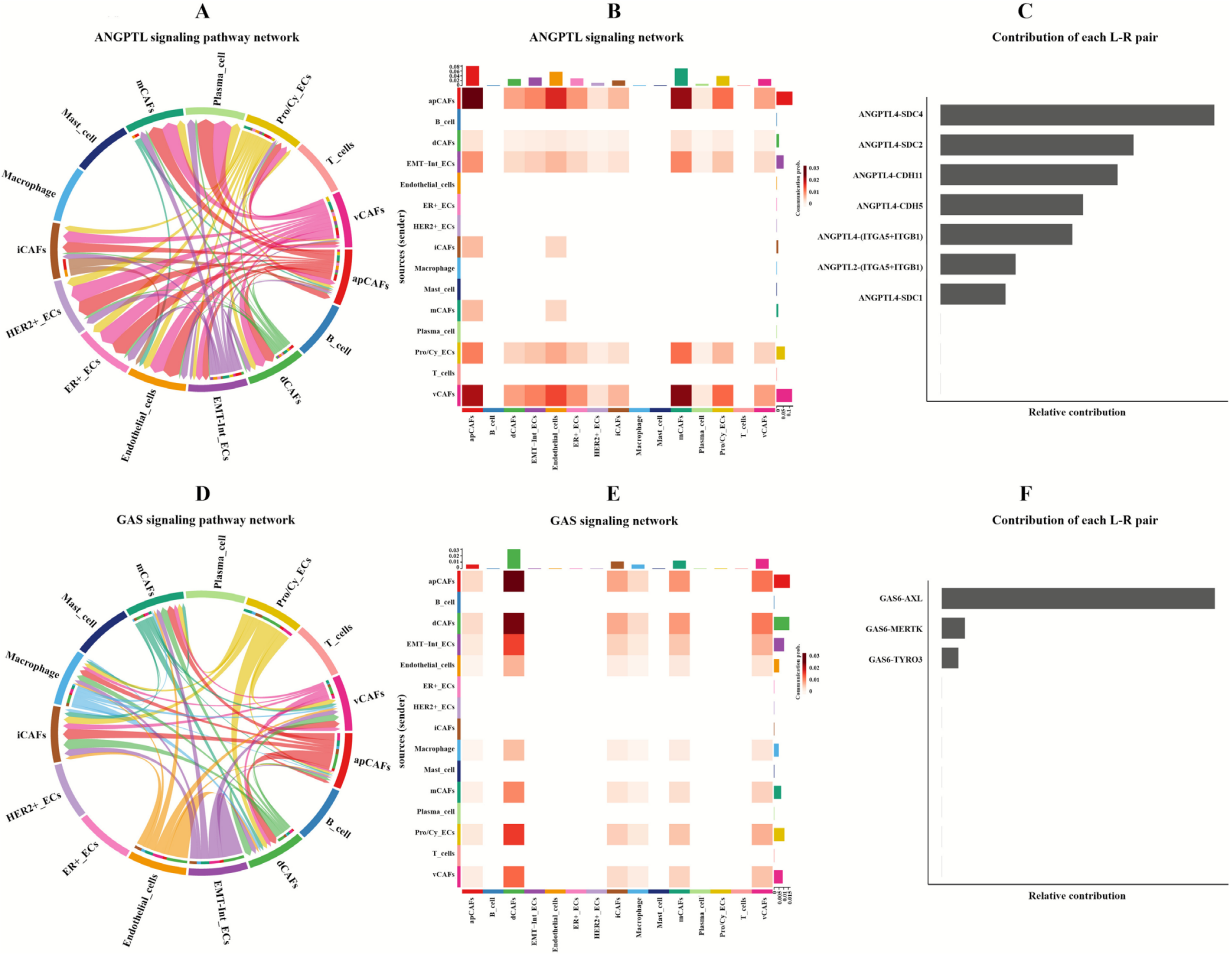
CellChat analysis of epithelial‐CAF crosstalk: ANGPTL pathway in ER+BC and GAS pathway in TNBC. (A) Chord diagram of ANGPTL pathway interactions in ER+BC. (B) Heatmap of ANGPTL pathway interactions in ER+BC. (C) Ranking of ligand‐receptor pair contributions in the ANGPTL pathway of ER+BC. (D) Chord diagram of GAS pathway interactions in TNBC. (E) Heatmap of GAS pathway interactions in TNBC. (F) Ranking of ligand‐receptor pair contributions in the GAS pathway of ER+BC.

Comparative analysis of subtype‐specific signaling pathways in BC (Figure S2A–C; Tables S4–, S6) revealed that ER+BC specimens exhibited distinct epithelial subpopulation signatures. Pro/Cy_ECs and EMT‐Int_ECs exclusively activated the ANGPTL pathway, while HER2+_ECs uniquely expressed SPP1 signaling. Comprehensive evaluation of pathway contribution hierarchies and communication of epithelial cells and CAFs (Figure 6A–C) identified the ANGPTL4‐SDC4 ligand‐receptor pair as exhibiting the highest pathway‐specific signaling activity among all interactions. Consequently, ANGPTL4‐SDC2 was established as the principal ANGPTL pathway regulator due to signaling capacity between epithelial cells and CAFs and documented role in tumor‐stroma crosstalk (Figure S3A).

In TNBC specimens, we identified compartmentalized GAS pathway activation (Figure 6D–F), with Pro/Cy_ECs and EMT‐Int_ECs clusters demonstrating robust GAS6‐AXL signaling (Figure S3B) and dCAFs exclusively expressing GAS6‐TYRO3. Spatial analysis revealed distinct paracrine signaling characteristics (Figure S3C). The experimental data demonstrated that the GAS6‐AXL/TYRO3 signaling axis functions as a critical regulator within this pathway, with mechanistic validation confirming its essential role in pathway modulation.

**FIGURE 7:**
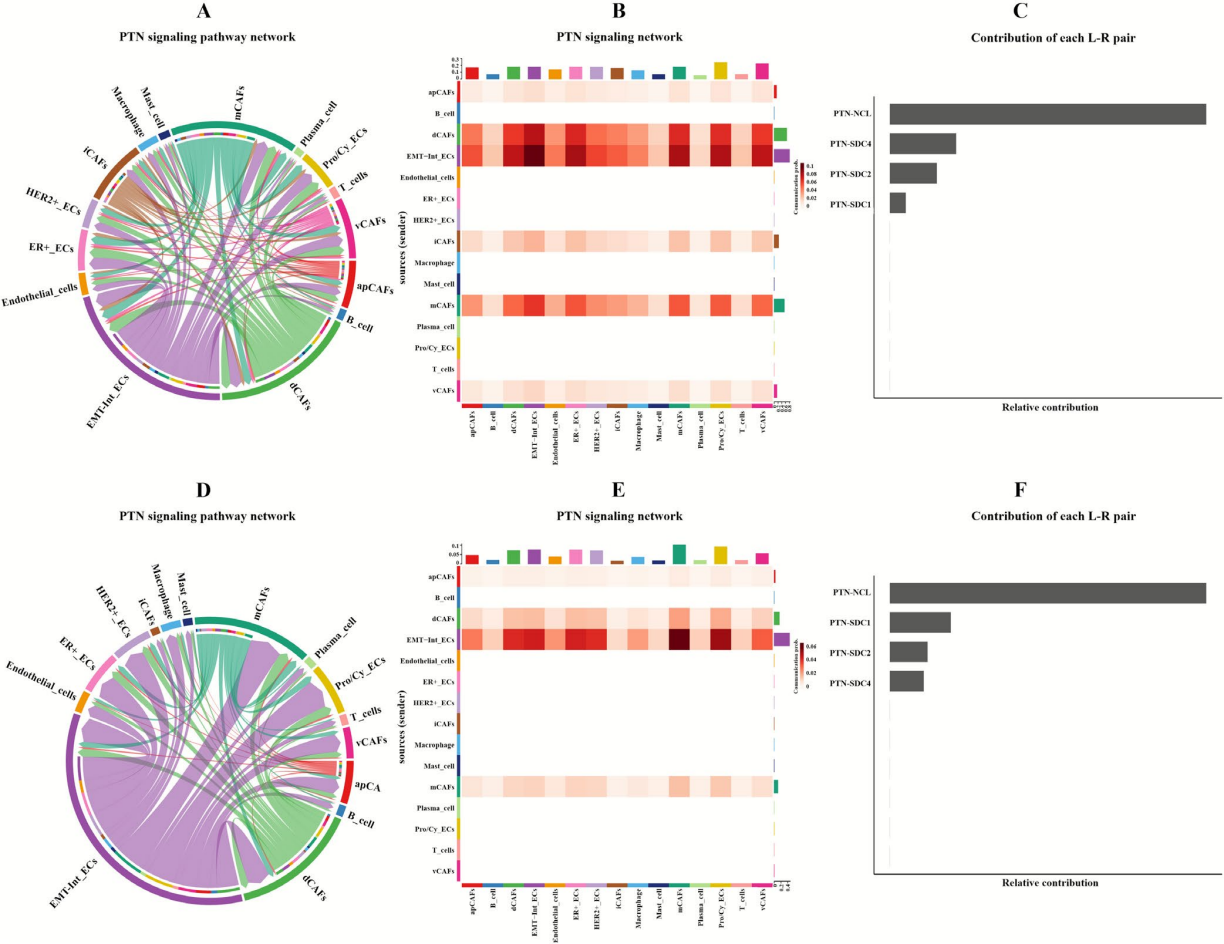
CellChat analysis of epithelial‐CAF crosstalk: PTN pathway in ER+BC and HER2+BC. (A) Chord diagram of PTN pathway interactions in ER+BC. (B) Heatmap of PTN pathway interactions in ER+BC. (C) Ranking of ligand‐receptor pair contributions in the PTN pathway of ER+BC. (D) Chord diagram of PTN pathway interactions in HER2+BC. (E) Heatmap of PTN pathway interactions in HER2+BC. (F) Ranking of ligand‐receptor pair contributions in the PTN pathway of HER2+BC.

Comparative signaling analysis revealed that while the EMT‐Int_ECs subpopulation uniquely activated the PTN pathway in both ER+BC and HER2+BC (Figure 7A–F), significant subtype‐specific differences emerged: PTN‐NCL was identified as the most significant ligand‐receptor pair in both BC subtypes. Notably, PTN‐SDC2 also exhibited strong interaction activity, with specific involvement in EMT‐Int_ECs‐CAFs crosstalk (Figure S3D,E). PTN signaling pathway exhibits higher activity levels in ER+BC compared to HER2+BC, while HER2+BC cases uniquely demonstrated EDN pathway activation through the EDN‐EDNRA axis.

### 3.7 SENIC Analysis of Epithelial Subpopulations

SENIC analysis of 5000 cells per BC subtype revealed ATF4, IRF1, and MAX as dominant transcriptional regulators in ER+BC Pro/Cy_ECs and EMT‐Int_ECs clusters (Figure S4A). Notably, ATF4 is highly expressed in CAFs (αSMA+) located in perivascular regions, and host ATF4 deficiency impairs the functions of CAFs at different stages of tumor development, thereby leading to a tumor‐suppressive phenotype [40]. The ANGPTL signaling pathway was predominantly mediated by ANGPTL4, which plays pivotal roles in vascular morphogenesis and stabilization. Particularly within tumor and inflammatory microenvironments, ANGPTL4 critically regulated vascular permeability and endothelial stability. Furthermore, ANGPTL4 had been well‐established as a significant biomarker, with extensive literature documenting its involvement in fibroblast activation and cancer progression [41]. Based on these findings, we proposed that ATF4 likely modulates tumor initiation and progression through transcriptional regulation of cell cycle and apoptosis‐related genes, consequently activating the ANGPTL signaling pathway.

Analysis of transcription factors in EMT‐Int_ECs of ER+BC and HER2+BC (Figure S4A,B) revealed that ELF5 activity is relatively higher in ER+BC. ELF5 expression was regulated by estrogen, which interacted with ELF5 to influence BC cell proliferation and survival. Additionally, studies showed that directly supplementing osteoblast cultures with estrogen increases PTN secretion [42]. In HER2+BC, the transcription factors ELF1, ELF2, and ELF3 exhibited higher activity. Studies indicated that among these, ELF3 primarily functions as a tumor suppressor in BC [43]. This gene not only promoted the formation of HER2+BC cells but also inhibited the proliferation of ER+BC cells [44]. Additionally, the differentiation and expression patterns of HER2 in neuroblastoma (NB) determine its cellular developmental potential, which is often associated with poor prognosis such as tumor metastasis. Meanwhile, PTN as a gene involved in neuronal differentiation, showed upregulated expression levels [45, 46].

Furthermore, we identified MEF2A (Figure S4C) among the transcription factors in TNBC, which is expressed in both Pro/Cy_ECs and EMT‐Int_ECs. MEF2A served as a key regulator of type I interferon induction [47]. Meanwhile, GAS6, the most crucial gene in the GAS pathway, can induce the secretion of type I interferon‐related cytokines and chemokines such as TGF‐β and TNF‐α. GAS6 not only played a pivotal role in tumor initiation, progression, and metastasis but also mediates drug resistance and immune evasion in tumors. In summary, our findings collectively demonstrated that the ANGPTL, PTN, and GAS signaling pathways play pivotal roles in mediating intercellular communication between epithelial cells and CAFs across different BC subtypes.

### 3.8 Macrophage Subtypes Classification and Developmental Trajectory Dynamics

**FIGURE 8:**
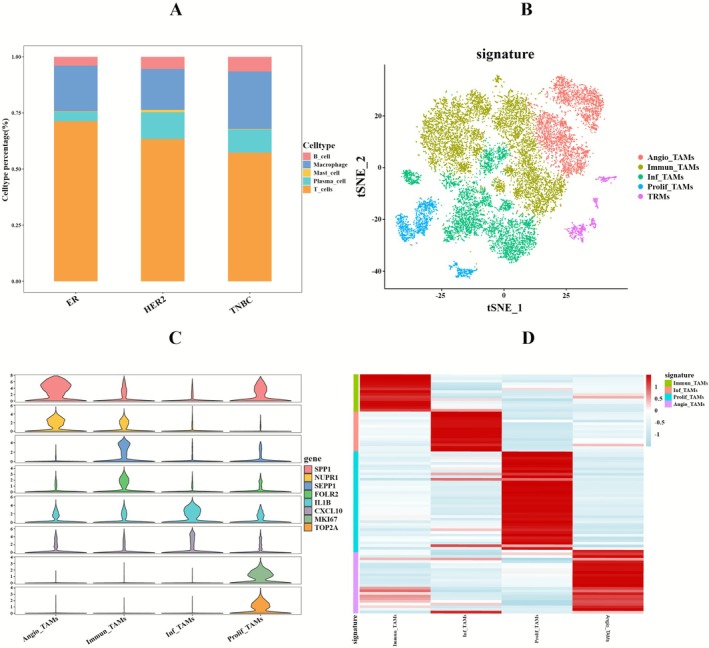
Immune cell activity assessment and macrophage polarization typing. (A) Bar plot showing immune cell proportions across breast cancer subtypes. (B) t‐SNE plot displays the distribution of macrophage subpopulations, labeled as Angio_TAMs, Immun_TAMs, Inf_TAMs, Prolif_TAMs, and TRMs. (C) Violin plot shows the marker genes for macrophage subpopulations. (D) Heatmap displays expression patterns of differentially expressed genes across subpopulations.

Analysis of the immune cell composition profile in the TME (Figure 8A) showed that T cells and macrophages predominated among the cell populations across all three BC subtypes. Comprehensive CellChat analysis of all cell types across BC subtypes demonstrated that macrophages exhibited the highest signaling interaction intensity among immune cell types in the TME across these subtypes (Figure S5A–C). The characterization employed the netAnalysis_signalingRole_scatter function.

Following strict quality control protocols, we isolated 16,324 macrophages for subsequent analysis. We then performed dimensionality reduction using t‐SNE and corrected for batch effects. First, we classified the macrophages into tissue‐resident macrophages (TRMs) and TAMs, based on differentially expressed genes identified in existing studies [48] (Figure S5D). Next, within the TAMs, we identified four distinct subpopulations based on unique marker gene expression profiles and their functional characteristics (Figure 8B): angiogenic TAMs (Angio_TAMs), marked by SPP1 and NUPR1; immunosuppressive TAMs (Immun_TAMs), marked by SEPP1 and FOLR2; inflammatory TAMs (Inf_TAMs), marked by IL1B and CXCL10; and proliferative TAMs (Prolif_TAMs), marked by MKI67 and TOP2A (Figure 8C). Heatmap analysis revealed significant differences in gene expression patterns among these subpopulations (Figure 8D).

**FIGURE 9:**
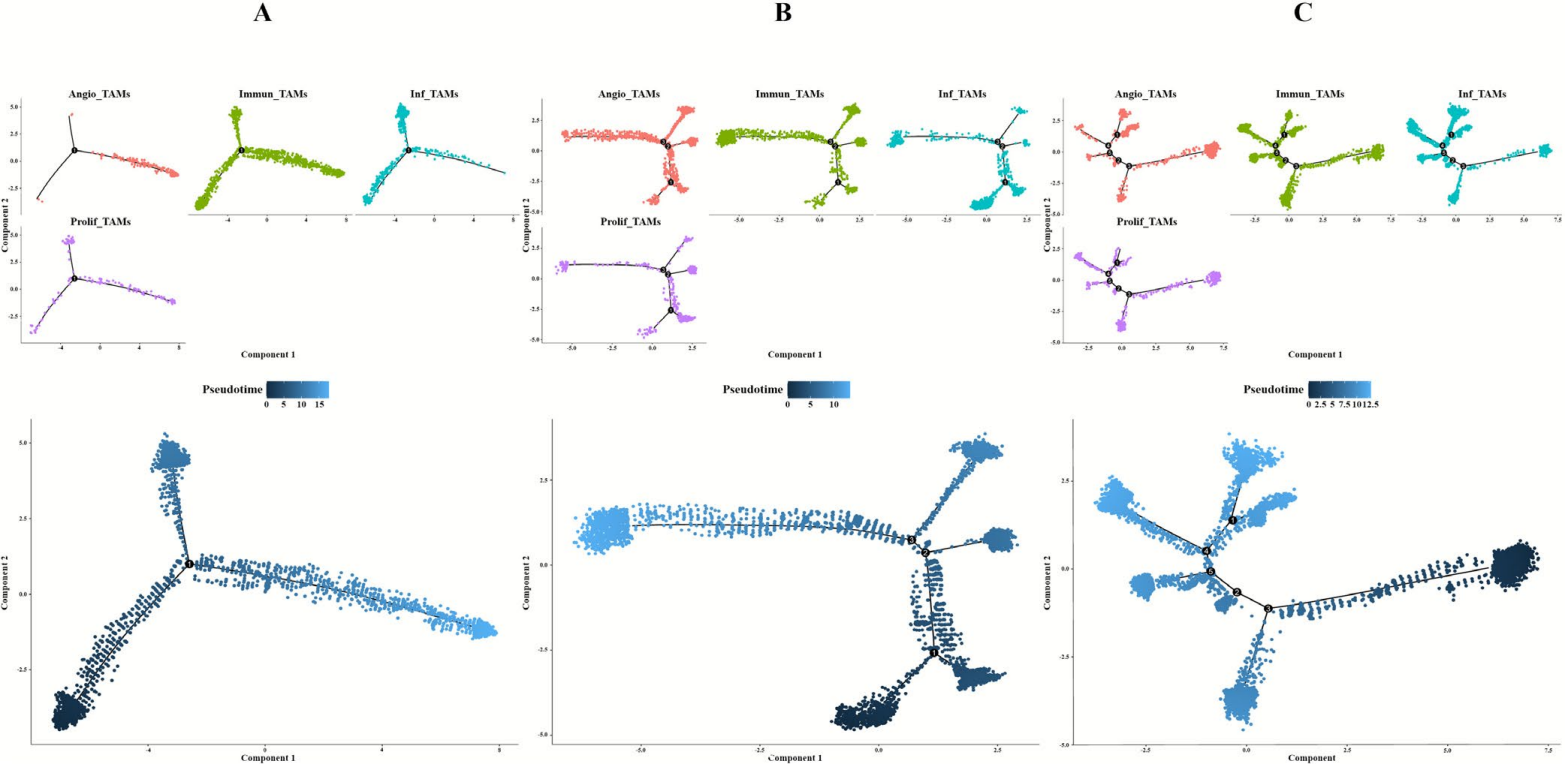
Pseudotime analysis of macrophage subpopulations. (A) Developmental trajectory and differentiation states of macrophage subpopulations in ER+BC. (B) Developmental trajectory and differentiation states of macrophage subpopulations in HER2+BC. (C) Developmental trajectory and differentiation states of macrophage subpopulations in TNBC.

Through Monocle based pseudotemporal trajectory analysis, we characterized TAMs differentiation dynamics across BC subtypes, revealed that: in ER+BC (Figure 9A), Immun_TAMs and Prolif_TAMs spanned the entire differentiation continuum while Inf_TAMs occupied early and mid‐term states and Angio_TAMs localized exclusively to terminal phases. In HER2+BC (Figure 9B), Immun_TAMs dominated intermediate‐late stages, Prolif_TAMs and Angio_TAMs clustered mid differentiation, and Inf_TAMs were restricted to initiation. In TNBC (Figure 9C), Prolif_TAMs persist throughout the differentiation of TAMs, and Angio_TAMs are abundant in the early stage, while Inf_TAMs and Immun_TAMs are located in the late stage of differentiation.

### 3.9 The Communication Interaction Between CAFs and TAMs

The preceding analysis demonstrated that CAFs are a critical cellular component in tumor progression. Meanwhile, TAMs as essential immune cells within the BC TME engage in biologically significant crosstalk with CAFs. These interactions play a pivotal role in tumorigenesis and disease progression. CellChat mediated analysis of intercellular communication networks revealed consistently robust interactions between CAFs and TAMs across BC subtypes, with distinct subtype‐specific patterns: ER+BC exhibited strongest signaling between Angio_TAMs and mCAFs (Figure S6A), HER2+BC showed maximal communication intensity between Inf_TAMs and mCAFs (Figure S6B), while TNBC demonstrated uniquely elevated crosstalk between Inf_TAMs and dCAFs (Figure S6C).

**FIGURE 10:**
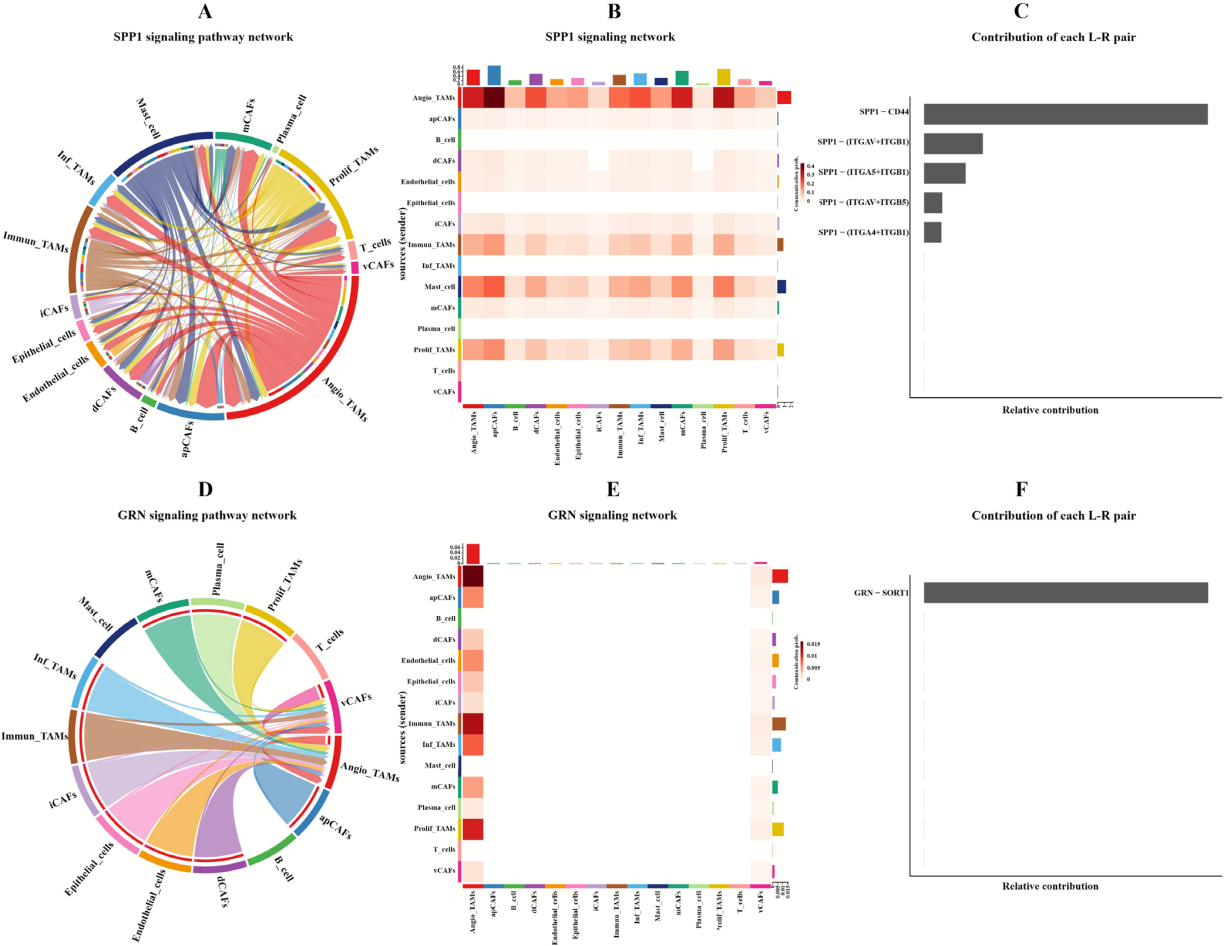
CellChat analysis of CAF‐TAM crosstalk in ER+BC. (A) Chord diagram of SPP1 pathway interactions in ER+BC. (B) Heatmap of SPP1 pathway interactions in ER+BC. (C) Ranking of ligand‐receptor pair contributions in the SPP1 pathway of ER+BC. (D) Chord diagram of GRN pathway interactions in ER+BC. (E) Heatmap of GRN pathway interactions in ER+BC. (F) Ranking of ligand‐receptor pair contributions in the GRN pathway of ER+BC.

Comprehensive ligand‐receptor profiling was performed by designating CAFs subpopulations as ligand sources and TAMs subpopulations as receptor targets. This systematic analysis identified distinct, subtype specific signaling architectures across BC subtypes (Figure S7A–C and Tables S7–, S9): in ER+BC, the SPP1 pathway showed significant activation in all fibroblast clusters except the low‐activity vCAFs (Figure 10A,B), with SPP1‐CD44 emerging as the dominant interaction (Figure S8A) among the key ligand‐receptor pairs SPP1‐CD44/(ITGAV+ITGB1)/(ITGA5+ITGB1) (Figure 10C). Notably, when Angio_TAMs functioned as autonomous receptors, specific GRN pathway enrichment (Figure 10D,E) occurred through the GRN‐SORT1 axis (Figures 10F and S8B), collectively highlighting the critical role of Angio_TAMs in ER+BC pathogenesis.

Among all subtypes, the SEMA3 signal is primarily involved in interactions between dCAFs, which exhibit the highest degree of variation among CAFs and TAMs. It is also expressed in mCAFs, but only in ER+BC (Figure S9A–F). In this context, SEMA3C‐(NRP1+NRP2) has been identified as the main ligand‐receptor pair (Figure S8C–E). Notably, in TNBC, dCAFs emit the strongest SEMA3C signal targeting TAMs, followed by ER+BC, while the signal is weakest in HER2+BC.

### 3.10 TIL Heterogeneity and Intercellular Communication in BC

The role of TILs varies significantly between BC subtypes. In this study, the relatively high proportion of TILs prompted us to focus our analysis on B cells and T cells. After strict quality control, we extracted 46,081 T cells and subjected them to standardized computational processing, including batch effect correction, dimensionality reduction via principal component analysis, and unsupervised clustering. This clustering identified three T cell subpopulations (Figure S10A): CD4_T (LTB, IL7R), CD8_T (GNLY, KLRD1), and Gamma‐Delta_T cells (MKI67, TYMS) (Figure S10B). Heatmap analysis shows significant differences in gene expression patterns among these subpopulations (Figure S10C).

The analysis of cell type activity for the three subtypes of BC using the CellChat analysis described in Figure S5 shows that in ER+BC, both B cells and T cells have low activity and are therefore excluded from further analysis. In HER2+BC, B cells exhibit high activity, while T cells still show very low activity. In TNBC, both B cells and T cells display high activity. Additionally, when examining other cell types, it was found that in HER2+BC, epithelial cells have the highest output signal intensity compared to other cell types, whereas in TNBC, fibroblasts exhibit the highest output signal intensity. Therefore, for CellChat interaction analysis, epithelial cells are used as ligands and B cells as receptors in HER2+BC. In TNBC, fibroblasts serve as ligands, while B cells and T cells are used as receptors.

**FIGURE 11:**
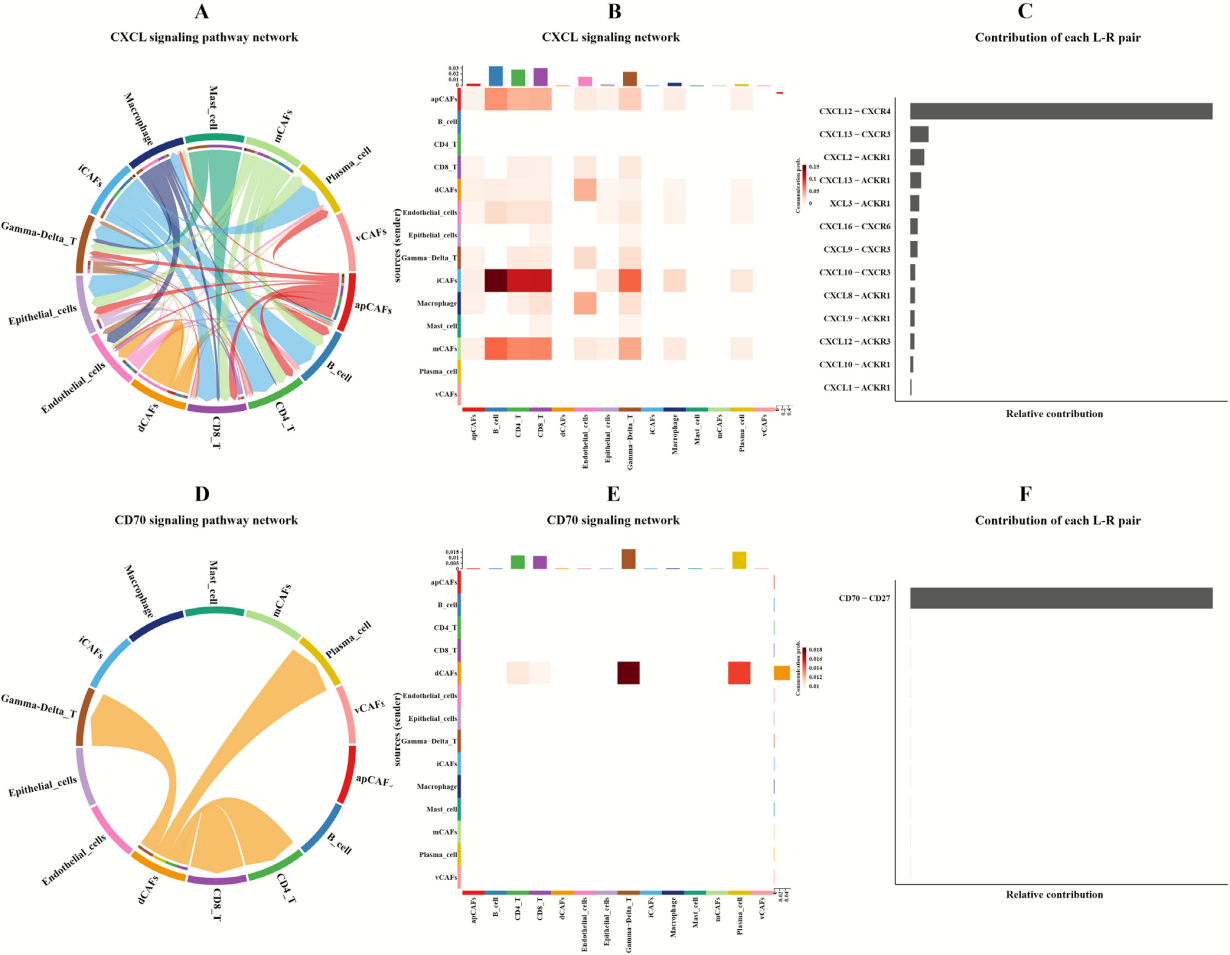
CellChat analysis of CAF‐TIL crosstalk in TNBC. (A) Chord diagram of CXCL pathway interactions in TNBC. (B) Heatmap of CXCL pathway interactions in TNBC. (C) Ranking of ligand‐receptor pair contributions in the CXCL pathway of TNBC. (D) Chord diagram of CD70 pathway interactions in TNBC. (E) Heatmap of CD70 pathway interactions in TNBC. (F) Ranking of ligand‐receptor pair contributions in the GRN pathway of CD70.

In HER2+BC (Figure S10D, Table S10), we observed that only EMT‐Int_ECs express the PTN signaling pathway. This finding is consistent with the interaction analysis between epithelial cells and CAFs, further supporting the role of PTN through EMT‐Int_ECs in HER2+BC. In TNBC (Figure S10E,F, Table S11), except for vCAFs, both B cells and T cells, when acting as receptors, exhibit strong expression of the CXCL signaling pathway (Figure 11A,B). The interaction analysis between CAFs and TAMs in TNBC also shows expression of the CXCL pathway, excluding vCAFs; this highlights the importance of the CXCL pathway in communication between CAFs and immune cells in TNBC, with the most significant ligand‐receptor pair being CXCL12‐CXCR4 (Figure 11C). Furthermore, due to the unique characteristics of dCAFs in TNBC, we found that when T cells act as receptors, dCAFs exclusively express the CD70 pathway (Figure 11D,E), with CD70‐CD27 as the key ligand‐receptor pair (Figure 11F).

### 3.11 Clinical Correlates From Bulk RNA‐Seq Data

In study, scRNA‐seq revealed that the signaling pathways involved in the interactions between epithelial cells and CAFs, between CAFs and TAMs, and among TILs, epithelial cells, and CAFs differ across various BC subtypes. To validate the role of these pathways in the different BC subtypes, we downloaded bulk RNA‐seq samples of 486 ER+BC, 30 HER2+BC, and 123 TNBC from TCGA. We then strengthened our conclusions by examining the correlation between key signaling molecules and clinical outcomes.

**FIGURE 12:**
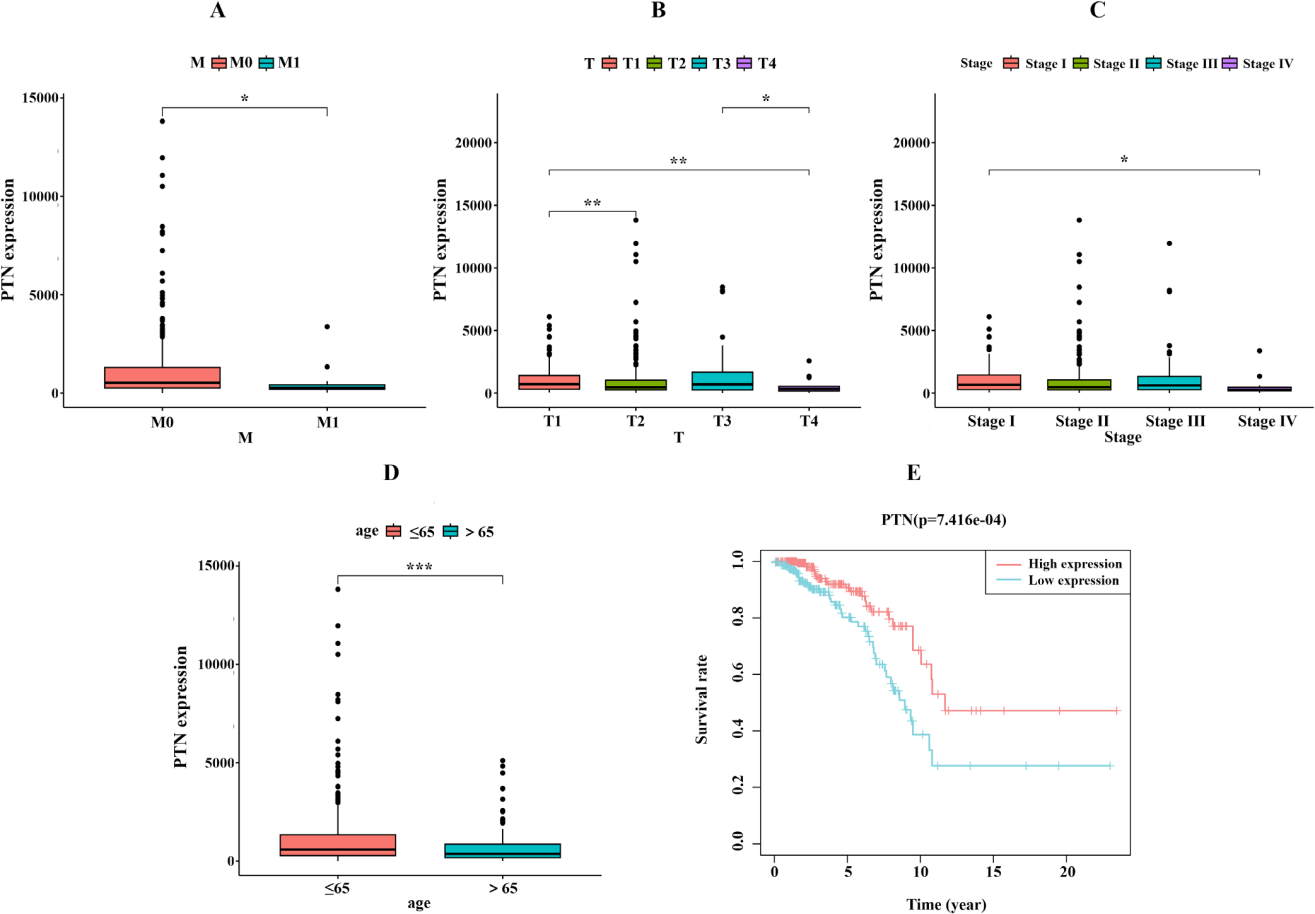
PTN expression and clinical outcomes in ER+BC. (A) PTN expression by M‐stage. (B) PTN expression by T‐stage. (C) PTN Expression by pathological stage. (D) PTN expression by age. (E) Kaplan–Meier survival analysis by PTN expression.

In ER+BC, the key gene PTN in the PTN pathway demonstrated strong clinical relevance, with its expression levels differing according to age, specifically whether patients are over 65 years old, as well as M stage, T stage, and overall stage (Figure 12A–D). Survival analysis indicated that high PTN expression correlates with higher survival rates, suggesting that PTN could be a potential therapeutic target in ER+BC (Figure 12E). Other key genes also exhibit clinical relevance. Similarly, in ER+BC, the key gene ANGPTL4 in the ANGPTL pathway shows differences in expression based on age (over or under 65 years) and Pathological Stage (Figure S11A,B). The key genes GRN in the GRN pathway and SPP1 in the SPP1 pathway show differences in expression related to T‐Stage, while the key gene SEMA3C in the SEMA3 pathway shows differences associated with age (Figure S11C–E). In TNBC, the key gene SEMA3C in the SEMA3 pathway show differences in Pathological Stage, respectively (Figure S11F).

## 4 Discussion

Multiple studies have confirmed that there are dynamic interactions among tumor cells, macrophages, fibroblasts, and TILs in BC, which collectively drive tumor progression [49]. While these four cell types are established contributors to cancer pathogenesis, their subtype‐specific prevalence and functional significance vary considerably across distinct BC subtypes. Despite their individual importance, the tripartite interactions among malignant epithelial cells, CAFs, TAMs, and TILs remain poorly characterized in ER+BC, HER2+BC, and TNBC. To address this knowledge gap, we systematically elucidated the functional relationships and collaborative mechanisms of these cell populations in different BC subtypes. This was achieved by analyzing scRNA‐seq data from the GEO database alongside bulk RNA‐seq data from the TCGA database.

BC progression is driven by both intrinsic factors in malignant epithelial cells and the TME [33]. Based on the integrated analysis, Pro/Cy_ECs, which are related to the cell cycle and proliferation, and EMT‐Int_ECs were selected as the primary study targets. Pathway enrichment analysis revealed significant activation of EMT and TNF−alpha Signaling via NF−κB in both clusters. EMT is well‐established as a critical driver of tumor invasion and metastasis across cancer types [50], with epithelial cells' dedifferentiation and accumulating mutations representing recognized hallmarks of cancer progression. The pro‐inflammatory cytokine TNF‐α demonstrates particular clinical relevance, as its signaling pathway correlates with increased tumor grade, metastatic frequency, and poor patient outcomes in BC [51]. Mechanistically, NF‐κB (as the primary transcriptional effector of TNF‐α signaling) has been shown to regulate ER activity [52]. Pro/Cy_ECs are mainly enriched in pathways related to the cell cycle and proliferation. In the context of tumors, the disruption of the balance between differentiation and proliferation is often accompanied by genomic instability, including CNV [53]. This may be related to the higher CNV scores observed in Pro/Cy_ECs compared to other subgroups. These findings strongly support Pro/Cy_ECs and EMT‐Int_ECs as the main epithelial cell subpopulations involved in breast tumorigenesis.

As critical components of the TME, fibroblasts mediate essential epithelial stromal crosstalk that drives BC progression. In the pseudotime analysis combining epithelial cells and CAFs, Pro/Cy ECs with high CNV scores, EMT‐Int ECs, and dCAFs subgroup are significantly clustered in the early differentiation stage of TNBC. In the pseudotime analysis of lung adenocarcinoma (LUAD), the trajectory is highly correlated with clinical staging. Cells in the early stages exhibit molecular characteristics associated with cell division and NK cell‐related activity [54]. These characteristics suggest that cells in the early differentiation stage have higher proliferative capacity and immune regulatory functions, such as modulation of NK cell activity, thereby playing a key role in tumor progression [54]. Collectively, these findings indicate that TNBC displays greater aggressiveness than other BC subtypes, as reflected by its molecular and cellular features.

This study employed CellChat to systematically map intercellular communication networks between epithelial cells and CAFs. To delineate subtype‐specific communication patterns that revealed exclusive activation of the ANGPTL signaling pathway in Pro/Cy_ECs and EMT‐Int_ECs clusters within ER+BC by conducting comparative analyses. Notably, recent functional studies have shown that pharmacological inhibition of ANGPTL4 produces: a dose‐dependent attenuation of EMT‐driven chemoresistance, disrupted tumor self‐organization in three‐dimensional (3D) models, significant reduction in metastatic capacity and impaired xenograft tumor growth. These collective observations strongly support the hypothesis that ANGPTL4 serves as a master regulator of ECM sensing, orchestrating phenotypic plasticity in both malignant cells and their stromal counterparts [41]. This is consistent with the analysis of scRNA‐seq and bulk RNA‐seq data, which suggest that ANGPTL4 is involved in fibroblast activation and tumor progression. Together, these findings identify the ANGPTL signaling pathway as a key contributor to the development of ER+BC, highlighting its potential therapeutic relevance.

In ER+BC and HER2+BC, the EMT‐Int_ECs subtype exhibits exclusive activation of the PTN pathway. However, the functional significance of PTN in the pathogenesis of human BC subtypes remains poorly understood. PTN has been implicated in tumorigenesis across multiple cancer types, including glioblastoma, melanoma, and pancreatic cancer. Its oncogenic effects are mediated through diverse signaling pathways, such as IRS‐1, AKT, ERK, STAT3, and Wnt, which collectively drive tumor proliferation, metastasis, and chemoresistance [55]. Notably, NCL expression is upregulated 3 to 6 fold in human BC cell lines compared to normal mammary epithelial cells [56], supporting its potential role in tumor progression. Interestingly, based on the SENIC method and bulk RNA‐seq analysis, we found that PTN signaling exerts an anti‐tumor effect in ER+BC by inhibiting the growth and invasion of tumor cells. In contrast, in HER2+BC, PTN promotes the proliferation of tumor cells. Overall, the PTN pathway is likely to play opposing roles in ER+BC and HER2+BC.

Comparative analysis reveals maximal GAS pathway activity in TNBC. The AXL‐GAS6 interaction and downstream activation are known to drive tumor proliferation and survival in multiple cancers, including prostate, colorectal, gastric, and renal carcinomas, as well as osteosarcoma [57]. In BC, AXL is highly expressed in TNBC cell lines and is associated with poor clinical outcomes [58], establishing GAS6‐AXL as a critical therapeutic target in TNBC.

Recent studies have shown that different macrophage subtypes play specific roles in tumors, making macrophage‐targeted therapy a promising immunotherapy strategy [59]. Through pseudotemporal analysis of TAM subtypes, it was found that Inf_TAMs are in the early differentiation stage in ER+BC and HER2+BC, while Inf_TAMs are in the late differentiation stage in TNBC. Notably, the differentiation of Angio_TAMs occurs in the early, middle, and late stages of TNBC, HER2+BC, and ER+BC, respectively. Inf_TAMs exert anti‐tumor effects by secreting pro‐inflammatory cytokines, such as TNF‐α and IL‐12, and by mediating cytotoxic effects on tumor cells [60]. Their differentiation is mainly driven by signals such as IFN‐γ. Moreover, Inf_TAMs antagonize Angio_TAMs and Immun_TAMs and can transdifferentiate into these subtypes. Therefore, the variations in TAM differentiation across BC subtypes reflect distinct immune response patterns in these cancers [60]. The differences in the interactions between CAFs and TAMs also reflect the varying roles of the TME in different BC subtypes.

In ER+BC, robust expression of the SPP1 signaling pathway was observed across most cellular populations. Angio_TAMs exhibited particularly strong activity. Studies of hormone receptor‐positive breast cancer (HR+BC) with high TILs infiltration demonstrate that increased SPP1^+^ macrophage populations significantly influence T‐cell activity [61]. However, the immunological impact of SPP1 appears context‐dependent. In ovarian cancer, for instance, elevated SPP1 expression correlates paradoxically with both increased TILs presence and upregulation of immune checkpoint markers, suggesting a potential role in fostering immune tolerance [62]. In the clinical analysis of the transcriptome of the SPP1 gene in ER+BC, we observed that its high expression promotes tumor growth, consistent with the conclusions of the aforementioned studies. Based on these observations, it is speculated that Angio_TAMs may regulate the activity of CAFs in ER+BC through the SPP1‐CD44 interaction, thereby promoting tumorigenesis.

GRN represents a critical pathway through which Angio_TAMs interact with CAFs in ER+BC. Previous studies have established associations between GRN (and its related genes) and the intrinsic immunogenicity of small cell lung cancer (SCLC). Notably, scRNA‐seq data demonstrate that GRN is highly expressed in a specific TAMs subpopulation [63]. Moreover, GRN has been identified as a tumor‐promoting factor in multiple malignancies, including skin, breast, astrocytoma, lung, ovarian, uterine, head and neck, liver, and hematologic cancers (lymphoma, multiple myeloma, and leukemia) [64]. Although there is currently no specific report on the enrichment of the GRN pathway in the Angio_TAMs subset of BC, GRN is significantly upregulated in the Angio_TAMs of ER+BC. Clinical transcriptome analysis shows that high expression of the GRN gene promotes tumor growth. Therefore, we propose that GRN regulates the function of CAFs in the TME influenced by this specific TAM population, which may promote tumor development.

The SEMA3 signaling pathway is mainly expressed in dCAFs of all BC subtypes. In BC, high expression of SEMA3C is negatively correlated with the differentiation level of tumor cells, with higher expression observed in less differentiated cells. Knockdown of SEMA3C can inhibit the proliferation, adhesion, and invasive ability of BC cells [65], indicating its pro‐tumorigenic role in BC. Moreover, our scRNA‐seq and bulk RNA‐seq analyses jointly indicate that the activity of the SEMA3 signaling pathway, reflected by increased gene expression, is upregulated in TNBC and ER+BC. Based on these analysis results, we reasonably speculate that the SEMA3 signaling pathway mainly exerts pro‐tumorigenic effects in TNBC and ER+BC.

Our research also indicates that there are significant differences in the activity of TILs between BC subtypes. Particularly in TNBC, TILs exhibit notable activity, with a focus on the CXCL and CD70 signaling pathways. CXCL, particularly CXCL12, promotes the recruitment of immunosuppressive cells. These include myeloid‐derived suppressor cells (MDSCs) that express CXCR4. These cells inhibit the anti‐tumor activity of TILs, thereby promoting immune escape [66]. CD70 has a comparable role in tumors. In renal cell carcinoma (RCC), there have been reports of persistent CD70‐CD27 signaling between tumor cells and TILs, suggesting that it may lead to increased malignancy [67]. Consequently, CXCL and CD70 jointly support tumor progression in TNBC by inhibiting the activity of TILs, making them promising targets for immunotherapy. Blocking this axis can restore TIL‐mediated anti‐tumor responses and enhance therapeutic efficacy.

Overall, this study analyzed cell–cell communication across different BC subtypes using scRNA‐seq data, focusing on the functional roles of specific cell‐type interactions within key signaling pathways. Clinical associations of relevant genes were further evaluated with bulk RNA‐seq data. In ER+BC, we not only confirmed that pathways such as ANGPTL, PTN, SPP1, and GRN influence tumor progression through distinct cellular subsets, but also identified ANGPTL and PTN as novel pathways active in this subtype. We further demonstrated the context‐dependent duality of the PTN pathway, which acts as a tumor suppressor in ER+BC while exerting an oncogenic role in HER2+BC. This finding provides new insight into tumor heterogeneity and context‐specific signaling regulation. In TNBC, in addition to characterizing the GAS and SEMA3 pathways, we first reported CD70 as a key pathway and a potential therapeutic target in this aggressive subtype. Collectively, these results deepen the mechanistic understanding of BC and offer a rationale for developing subtype‐specific precision therapies. A major limitation of this work is the lack of experimental validation. Although our computational approach provides comprehensive bioinformatic insights, it carries inherent limitations. Future studies should therefore prioritize multimodal experimental validation, including clinical‐correlative studies, to substantiate these findings and establish their biological and translational relevance.

## Author Contributions

**Yunlong Zhao:** conceptualization, data curation, formal analysis, visualization, software, writing – original draft. **Xiaoyu Zhang:** visualization, validation, investigation. **Yingying Wang, Xiaomin Yu, and Fengchun Lv:** validation, investigation. **Mingyu Gong:** visualization, methodology, project administration, writing – original draft. **Xiu‐An Yang:** conceptualization, methodology, funding acquisition, writing – review and editing. All authors have read and approved the final version of the manuscript.

## Funding

This work was supported by Natural Science Foundation of Hebei Province (Grant Number H2020406049) and Initial Scientific Research Fund for High‐Level Talents of Chengde Medical University (Grant Number 201901).

## Ethics Statement

The authors have nothing to report.

## Consent

The authors have nothing to report.

## Conflicts of Interest

The authors declare no conflicts of interest.

## Supporting information


**Figure S1:** InferCNV analysis results of epithelial cells and fibroblasts. (A) Heatmap shows the chromosomal mapping of single‐cell large‐scale CNVs in epithelial cells inferred through scRNA‐seq. (B) Boxplot displaying CNV scores of epithelial cells. (C) Heatmap shows the chromosomal mapping of single‐cell large‐scale CNVs in fibroblasts inferred through scRNA‐seq. (D) Boxplot displaying CNV scores of fibroblasts.

**Figure S2:** Diagram of ligand‐receptor mediated epithelial‐CAF interactions across BC subtypes. (A) Diagram of ligand‐receptor mediated epithelial‐CAF interactions in ER+BC. (B) Diagram of ligand‐receptor mediated epithelial‐CAF interactions in HER2+BC. (C) Diagram of ligand‐receptor mediated epithelial‐CAF interactions in TNBC.

**Figure S3:** Key ligand‐receptor pairs mediating epithelial‐CAF interactions. (A) Circle plot illustrating intercellular communication mediated by ANGPTL4‐SDC2 in ER+BC. (B) Circle plot illustrating intercellular communication mediated by GAS6‐AXL in TNBC. (C) Circle plot illustrating intercellular communication mediated by GAS6‐TYRO3 in TNBC. (D) Circle plot illustrating intercellular communication mediated by PTN‐SDC2 in ER+BC. (E) Circle plot illustrating intercellular communication mediated by PTN‐SDC2 in HER2+BC.

**Figure S4:** SCENIC analysis of epithelial subpopulations across BC subtypes. (A) Heatmap displaying the top 50 most active transcription factors in epithelial subpopulations of ER+BC. (B) Heatmap displaying the top 50 most active transcription factors in epithelial subpopulations of HER2+BC. (C) Heatmap displaying the top 50 most active transcription factors in epithelial subpopulations of TNBC.

**Figure S5:** CellChat analysis of cellular activity across BC subtypes and differential expression in TAMs and TRMs. (A) Activity of cell types in ER+BC by CellChat analysis. (B) Activity of cell types in HER2+BC by CellChat analysis. (C) Activity of cell types in TNBC by CellChat analysis. (D) Heatmap displays expression patterns of differentially expressed genes in TAMs and TRMs.

**Figure S6:** Cell‐to‐cell interaction analysis between CAF and TAM subtypes in the tumor microenvironment using CellChat. (A) CellChat analysis for ER+BC samples shows that Angio_TAMs and mCAFs subtypes exhibit the highest level of communication among the cell subtypes within the tumor microenvironment. (B) In HER2+BC samples, the Inf_TAMs and mCAFs subtypes are identified as having the most active cell‐to‐cell interactions. (C) In TNBC, Inf_TAMs and dCAFs subtypes demonstrate the most significant cell‐to‐ cell interactions.

**Figure S7:** Diagram of ligand‐receptor mediated CAF‐TAM interactions across BC subtypes. (A) Diagram of ligand‐receptor mediated CAF‐TAM interactions in ER+BC. (B) Diagram of ligand‐receptor mediated CAF‐TAM interactions in HER2+BC. (C) Diagram of ligand‐receptor mediated CAF‐TAM interactions in TNBC.

**Figure S8:** Key ligand‐receptor pairs mediating CAF‐TAM interactions. (A) Circle plot illustrating intercellular communication mediated by SPP1‐CD44. (B) Circle plot illustrating intercellular communication mediated by GRN‐SORT1. (C) Circle plot illustrating intercellular communication mediated by SEMA3C‐ (NRP1+NRP2) in ER+BC. (D) Circle plot illustrating intercellular communication mediated by SEMA3C‐(NRP1+NRP2) in HER2+BC. (E) Circle plot illustrating intercellular communication mediated by SEMA3C‐(NRP1+NRP2) in TNBC.

**Figure S9:** CellChat analysis of CAF‐TAM crosstalk: SEMA3 pathway expressed in dCAFs. (A) Chord diagram of SEMA3 pathway interactions in ER+BC. (B) Chord diagram of SEMA3 pathway interactions in HER2+BC. (C) Chord diagram of SEMA3 pathway interactions in TNBC. (D) Heatmap of SEMA3 pathway interactions in ER+BC. (E) Heatmap of SEMA3 pathway interactions in HER2+BC. (F) Heatmap of SEMA3 pathway interactions in TNBC.

**Figure S10:** Analysis of T‐cell subsets and their ligand‐receptor mediated interactions with epithelial cells and fibroblasts across BC subtypes. (A) t‐SNE plot displays the distribution of T cell subpopulations, labeled as CD4_T, CD8_T, Gamma‐Delta_T. (B) Violin plot shows the marker genes for T cell subpopulations. (C) Heatmap displays expression patterns of differentially expressed genes across subpopulations. (D) Diagram of ligand‐receptor mediated epithelial‐B cell interactions in HER2+BC. (E) Diagram of ligand‐receptor mediated CAF‐B cell interactions in TNBC. (F) Diagram of ligand‐receptor mediated CAF‐T cell interactions in TNBC.

**Figure S11:** Clinical relevance of key genes from CellChat‐derived ligand‐receptor pairs. (A) ANGPTL4 expression by age in ER+BC. (B) ANGPTL4 expression by pathological stage in ER+BC. (C) GRN expression by T‐stage. (D) SEMA3C expression by age in ER+BC. (E) SPP1 expression by T‐stage in ER+BC. (F) SEMA3C expression by pathological stage in TNBC.

**Table S1:** Detailed information of the patients enrolled in this study.

**Table S2:** Pathways enriched by marker genes of epithelial cell subsets.

**Table S3:** Pathways enriched by marker genes of fibroblast subsets.

**Table S4:** Signaling pathways between epithelial cells and CAFs in ER+BC.

**Table S5:** Signaling pathways between epithelial cells and CAFs in HER2+BC.

**Table S6:** Signaling pathways between epithelial cells and CAFs in TNBC.

**Table S7:** Signaling pathways between CAFs and TAMs in ER+BC.

**Table S8:** Signaling pathways between CAFs and TAMs in HER2+ BC.

**Table S9:** Signaling pathways between CAFs and TAMs in TNBC.

**Table S10:** Signaling pathways between epithelial cells and B cells in HER2+BC.

**Table S11:** Signaling pathways between CAFs and B cells/T cells in TNBC.

## Data Availability

The single‐cell RNA sequencing datasets of primary breast cancer were obtained from the Gene Expression Omnibus (GEO, https://www.ncbi.nlm.nih.gov/gds/?term=) using accession numbers GSE176078, GSE248288, GSE161529, and GSE180286. Additionally, bulk RNA sequencing data for breast cancer were obtained from The Cancer Genome Atlas (TCGA, https://portal.gdc.cancer.gov).
