# Supplementary figures and images for: Single‐Cell RNA Sequencing and Bulk RNA Sequencing Revealed the Interplay Between Intratumoral Heterogeneity and the Tumor Microenvironment in Breast Cancer
Source: Cancer Med. 2026 Feb 3;15(2):e71600. doi: 10.1002/cam4.71600 (PMC12868385; doi:10.1002/cam4.71600)

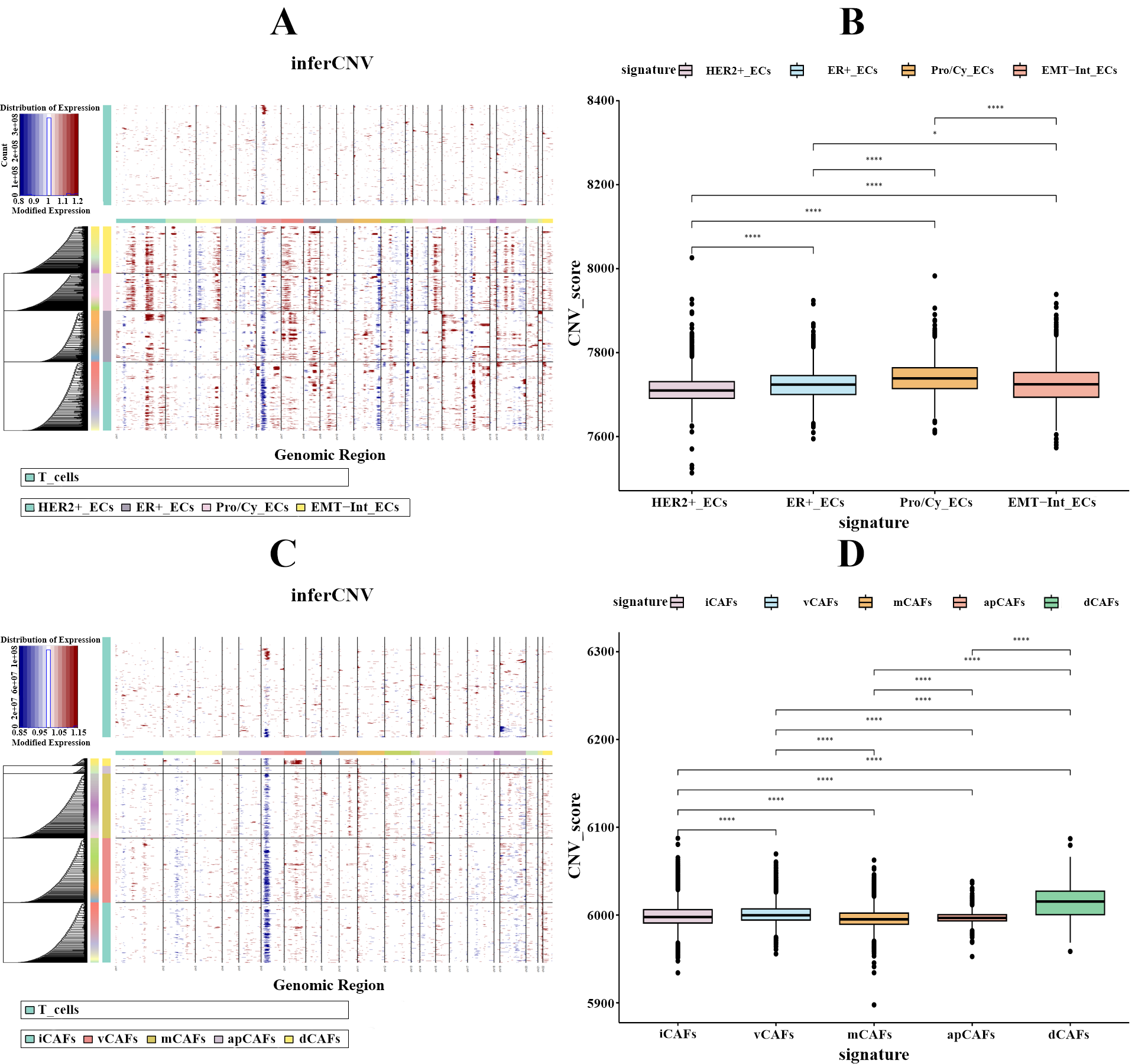

Supplement: Supplementary file 1 — Figure S1: InferCNV analysis results of epithelial cells and fibroblasts. (A) Heatmap shows the chromosomal mapping of single‐cell large‐scale CNVs in epithelial cells inferred through scRNA‐seq. (B) Boxplot displaying CNV scores of epithelial cells. (C) Heatmap shows the chromosomal mapping of single‐cell large‐scale CNVs in fibroblasts inferred through scRNA‐seq. (D) Boxplot displaying CNV scores of fibroblasts. [file CAM4-15-e71600-s017.png]

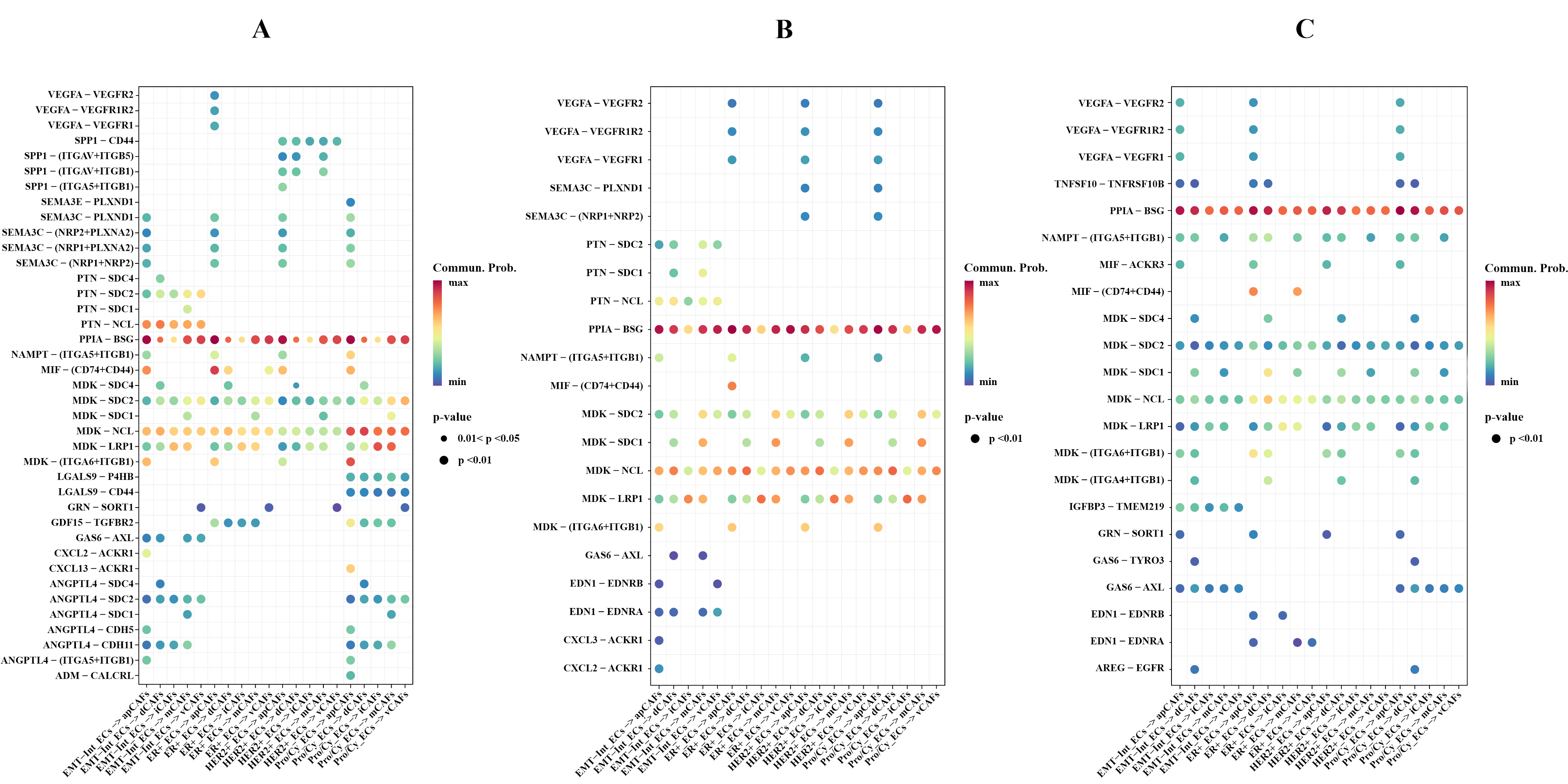

Supplement: Supplementary file 2 — Figure S2: Diagram of ligand‐receptor mediated epithelial‐CAF interactions across BC subtypes. (A) Diagram of ligand‐receptor mediated epithelial‐CAF interactions in ER+BC. (B) Diagram of ligand‐receptor mediated epithelial‐CAF interactions in HER2+BC. (C) Diagram of ligand‐receptor mediated epithelial‐CAF interactions in TNBC. [file CAM4-15-e71600-s018.png]

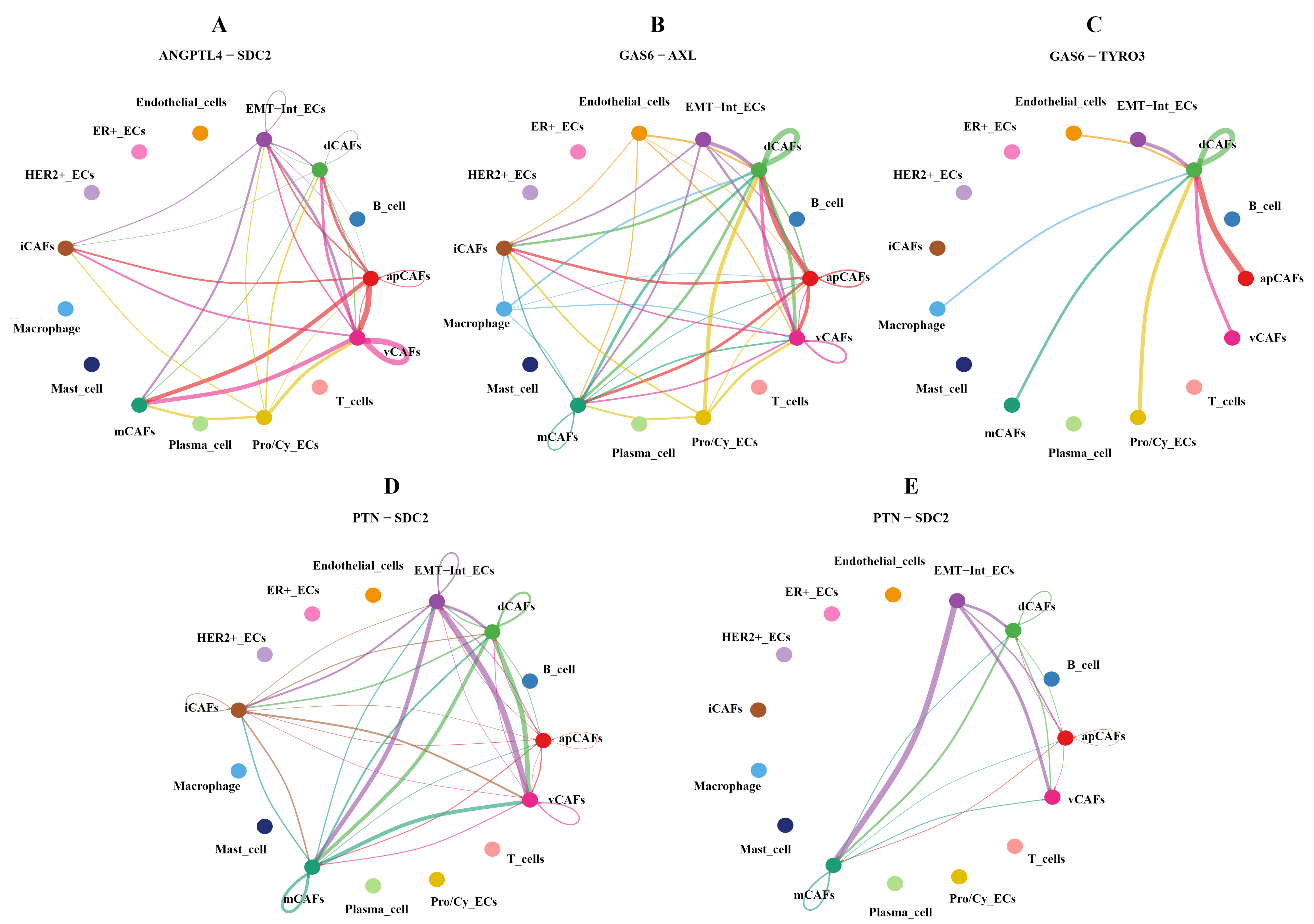

Supplement: Supplementary file 3 — Figure S3: Key ligand‐receptor pairs mediating epithelial‐CAF interactions. (A) Circle plot illustrating intercellular communication mediated by ANGPTL4‐SDC2 in ER+BC. (B) Circle plot illustrating intercellular communication mediated by GAS6‐AXL in TNBC. (C) Circle plot illustrating intercellular communication mediated by GAS6‐TYRO3 in TNBC. (D) Circle plot illustrating intercellular communication mediated by PTN‐SDC2 in ER+BC. (E) Circle plot illustrating intercellular communication mediated by PTN‐SDC2 in HER2+BC. [file CAM4-15-e71600-s021.png]

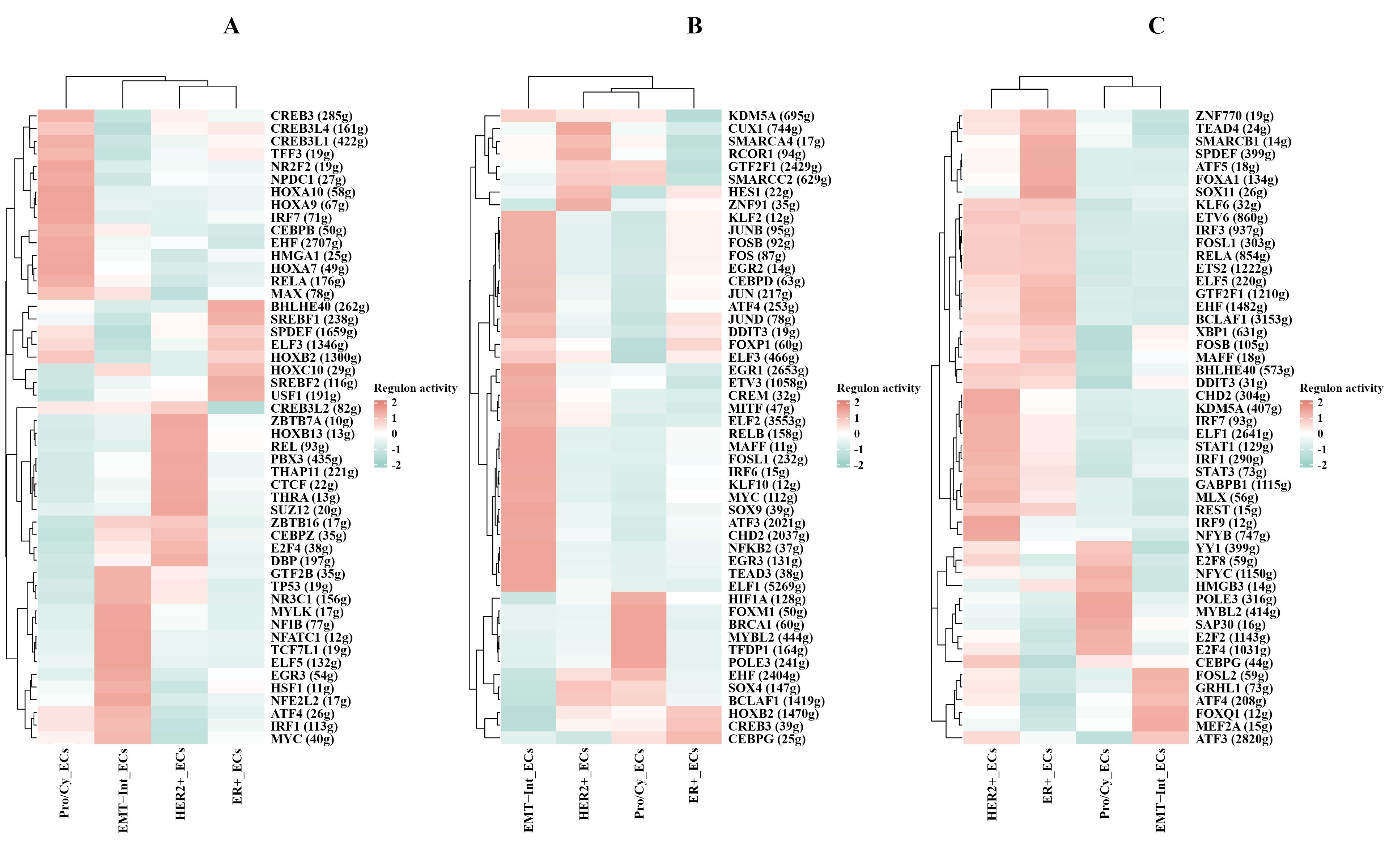

Supplement: Supplementary file 4 — Figure S4: SCENIC analysis of epithelial subpopulations across BC subtypes. (A) Heatmap displaying the top 50 most active transcription factors in epithelial subpopulations of ER+BC. (B) Heatmap displaying the top 50 most active transcription factors in epithelial subpopulations of HER2+BC. (C) Heatmap displaying the top 50 most active transcription factors in epithelial subpopulations of TNBC. [file CAM4-15-e71600-s001.png]

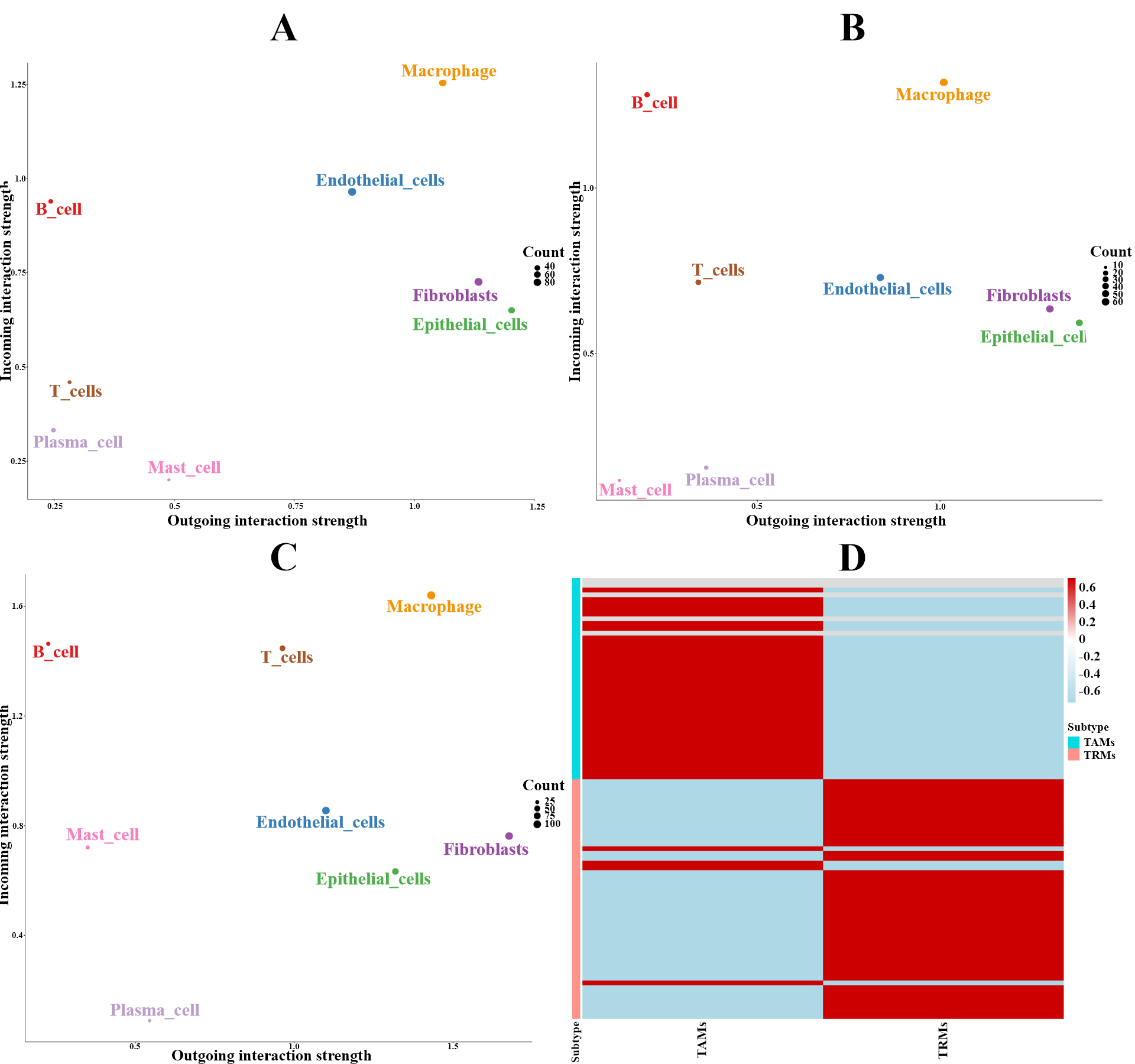

Supplement: Supplementary file 5 — Figure S5: CellChat analysis of cellular activity across BC subtypes and differential expression in TAMs and TRMs. (A) Activity of cell types in ER+BC by CellChat analysis. (B) Activity of cell types in HER2+BC by CellChat analysis. (C) Activity of cell types in TNBC by CellChat analysis. (D) Heatmap displays expression patterns of differentially expressed genes in TAMs and TRMs. [file CAM4-15-e71600-s008.png]

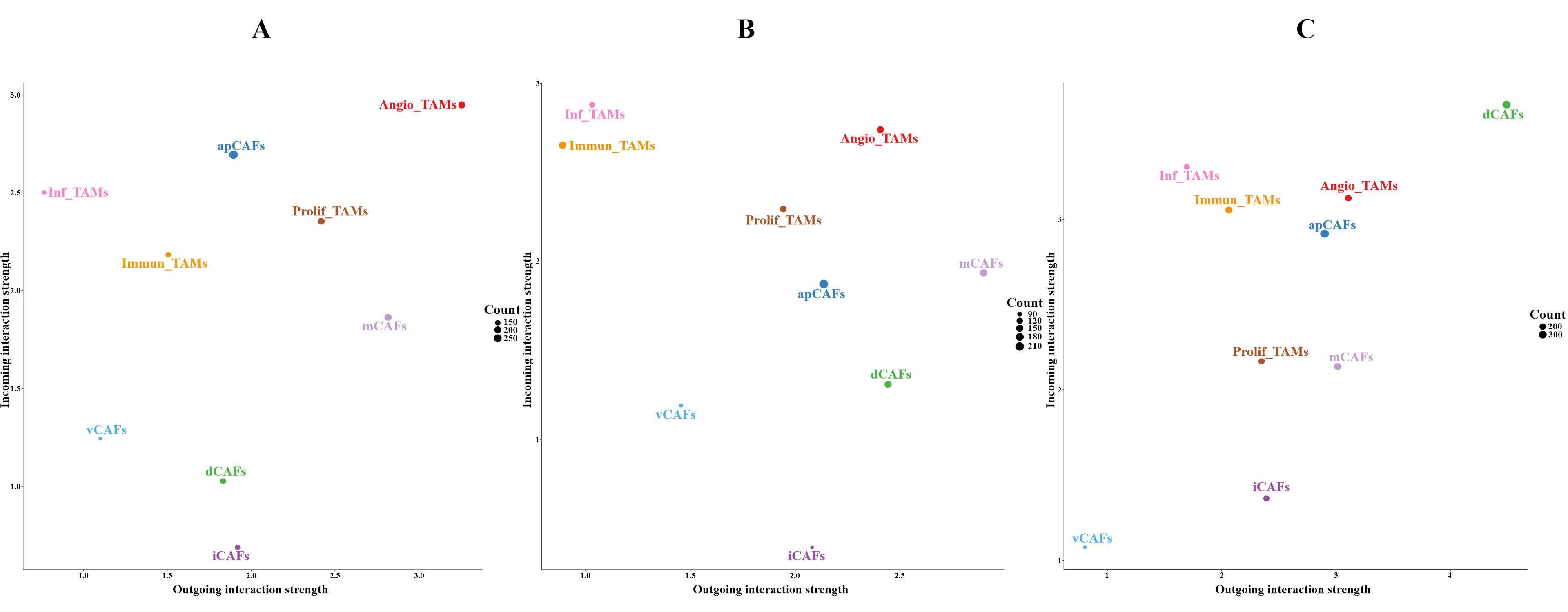

Supplement: Supplementary file 6 — Figure S6: Cell‐to‐cell interaction analysis between CAF and TAM subtypes in the tumor microenvironment using CellChat. (A) CellChat analysis for ER+BC samples shows that Angio_TAMs and mCAFs subtypes exhibit the highest level of communication among the cell subtypes within the tumor microenvironment. (B) In HER2+BC samples, the Inf_TAMs and mCAFs subtypes are identified as having the most active cell‐to‐cell interactions. (C) In TNBC, Inf_TAMs and dCAFs subtypes demonstrate the most significant cell‐to‐ cell interactions. [file CAM4-15-e71600-s004.png]

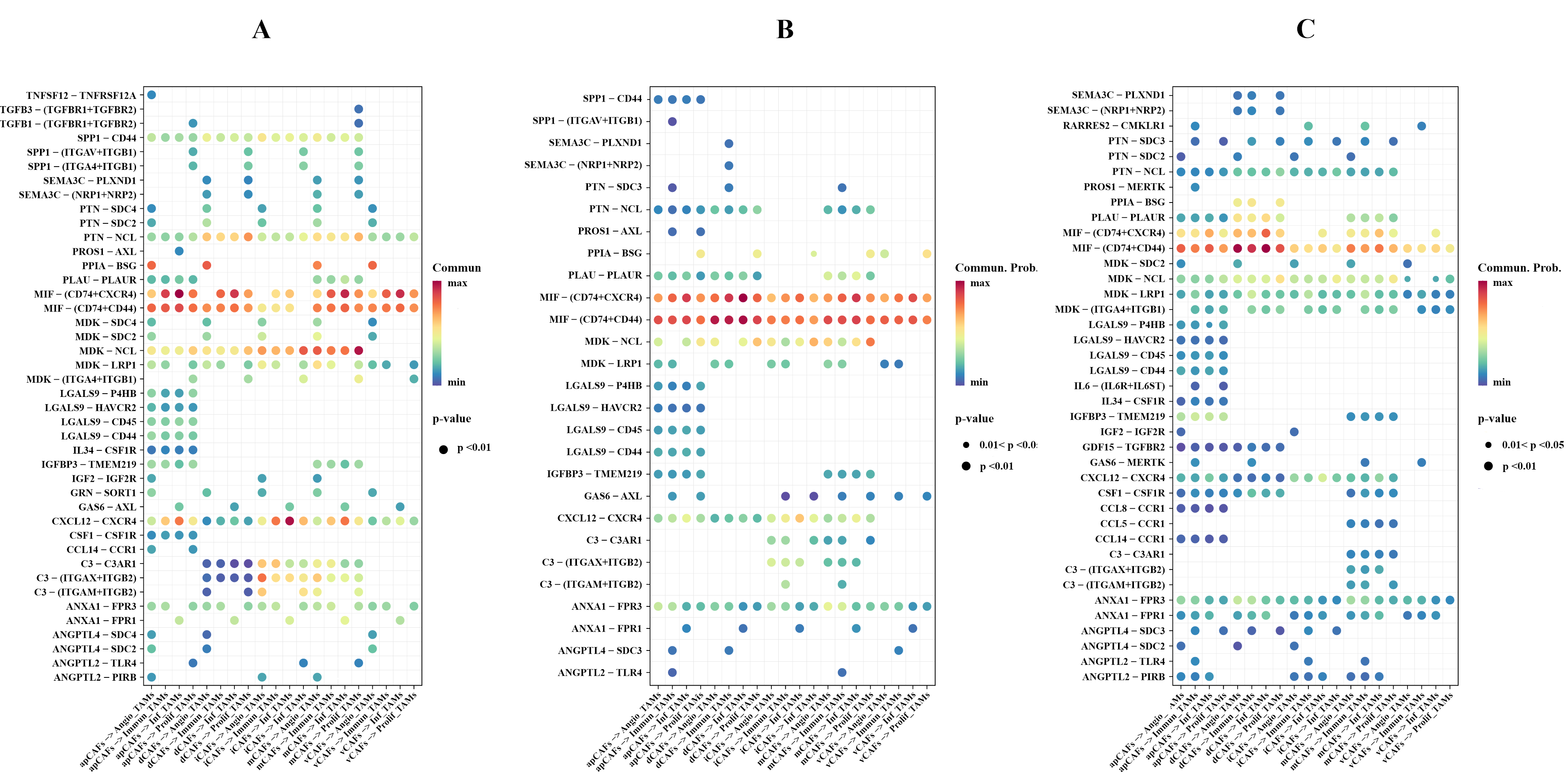

Supplement: Supplementary file 7 — Figure S7: Diagram of ligand‐receptor mediated CAF‐TAM interactions across BC subtypes. (A) Diagram of ligand‐receptor mediated CAF‐TAM interactions in ER+BC. (B) Diagram of ligand‐receptor mediated CAF‐TAM interactions in HER2+BC. (C) Diagram of ligand‐receptor mediated CAF‐TAM interactions in TNBC. [file CAM4-15-e71600-s011.png]

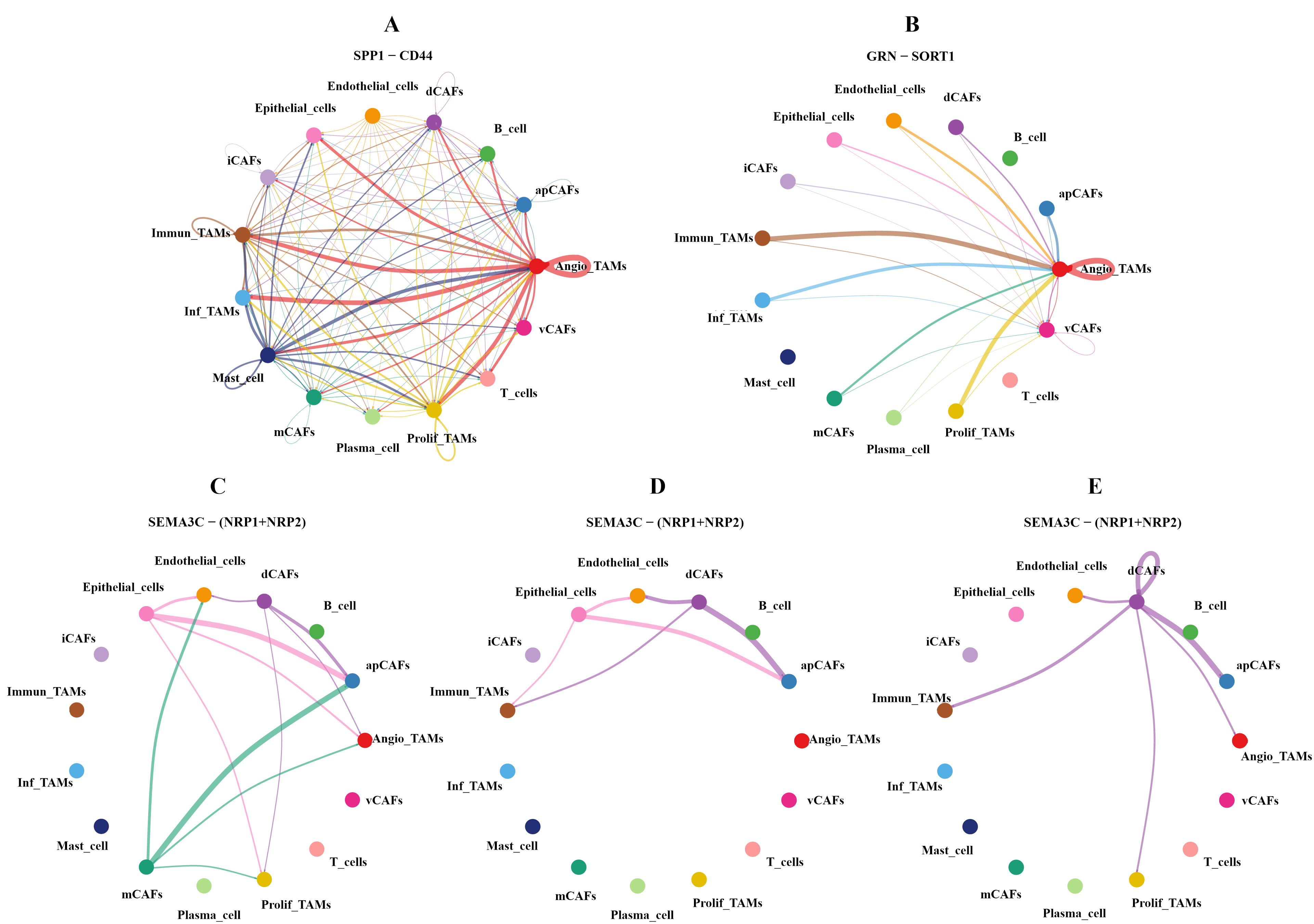

Supplement: Supplementary file 8 — Figure S8: Key ligand‐receptor pairs mediating CAF‐TAM interactions. (A) Circle plot illustrating intercellular communication mediated by SPP1‐CD44. (B) Circle plot illustrating intercellular communication mediated by GRN‐SORT1. (C) Circle plot illustrating intercellular communication mediated by SEMA3C‐ (NRP1+NRP2) in ER+BC. (D) Circle plot illustrating intercellular communication mediated by SEMA3C‐(NRP1+NRP2) in HER2+BC. (E) Circle plot illustrating intercellular communication mediated by SEMA3C‐(NRP1+NRP2) in TNBC. [file CAM4-15-e71600-s003.png]

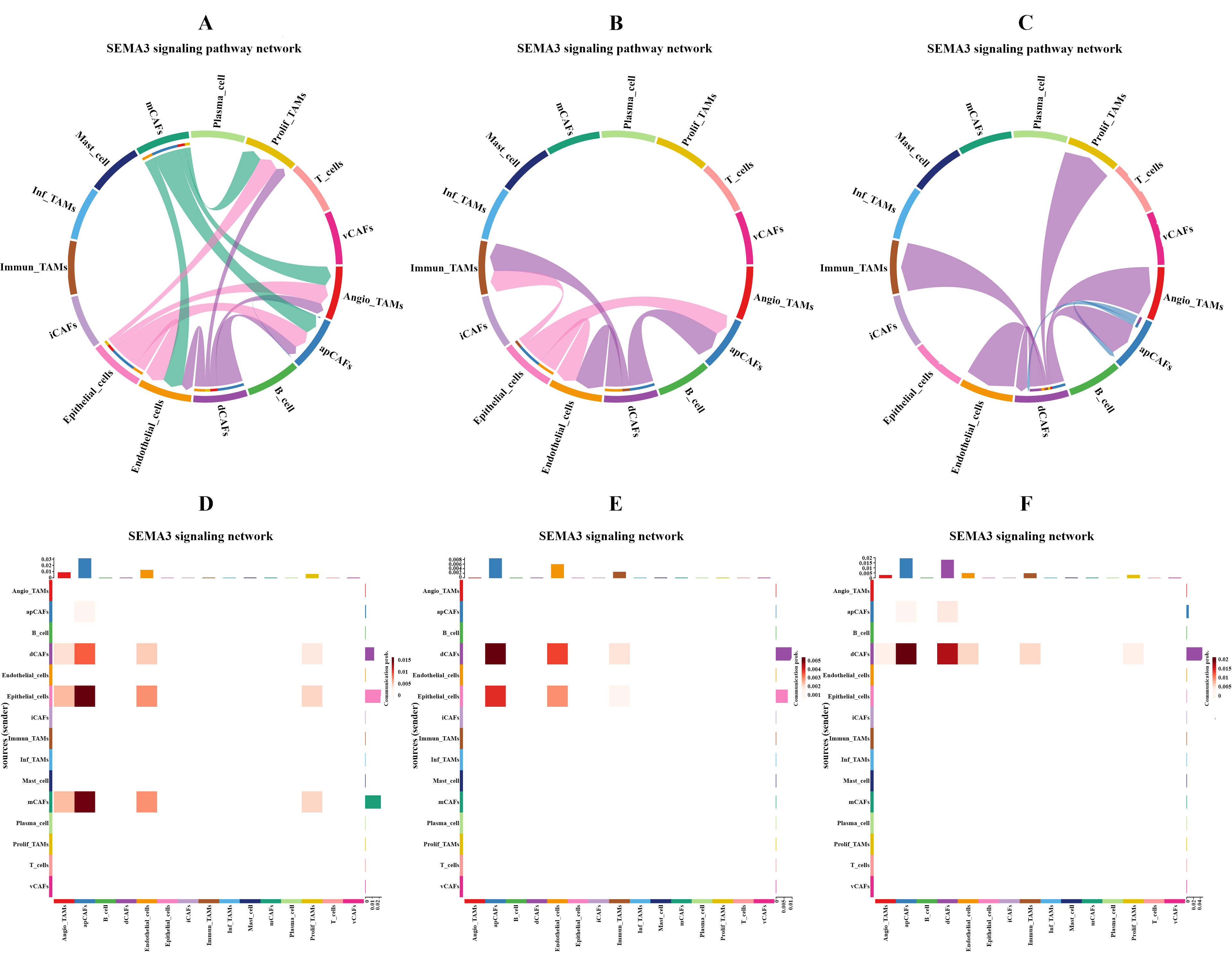

Supplement: Supplementary file 9 — Figure S9: CellChat analysis of CAF‐TAM crosstalk: SEMA3 pathway expressed in dCAFs. (A) Chord diagram of SEMA3 pathway interactions in ER+BC. (B) Chord diagram of SEMA3 pathway interactions in HER2+BC. (C) Chord diagram of SEMA3 pathway interactions in TNBC. (D) Heatmap of SEMA3 pathway interactions in ER+BC. (E) Heatmap of SEMA3 pathway interactions in HER2+BC. (F) Heatmap of SEMA3 pathway interactions in TNBC. [file CAM4-15-e71600-s015.png]

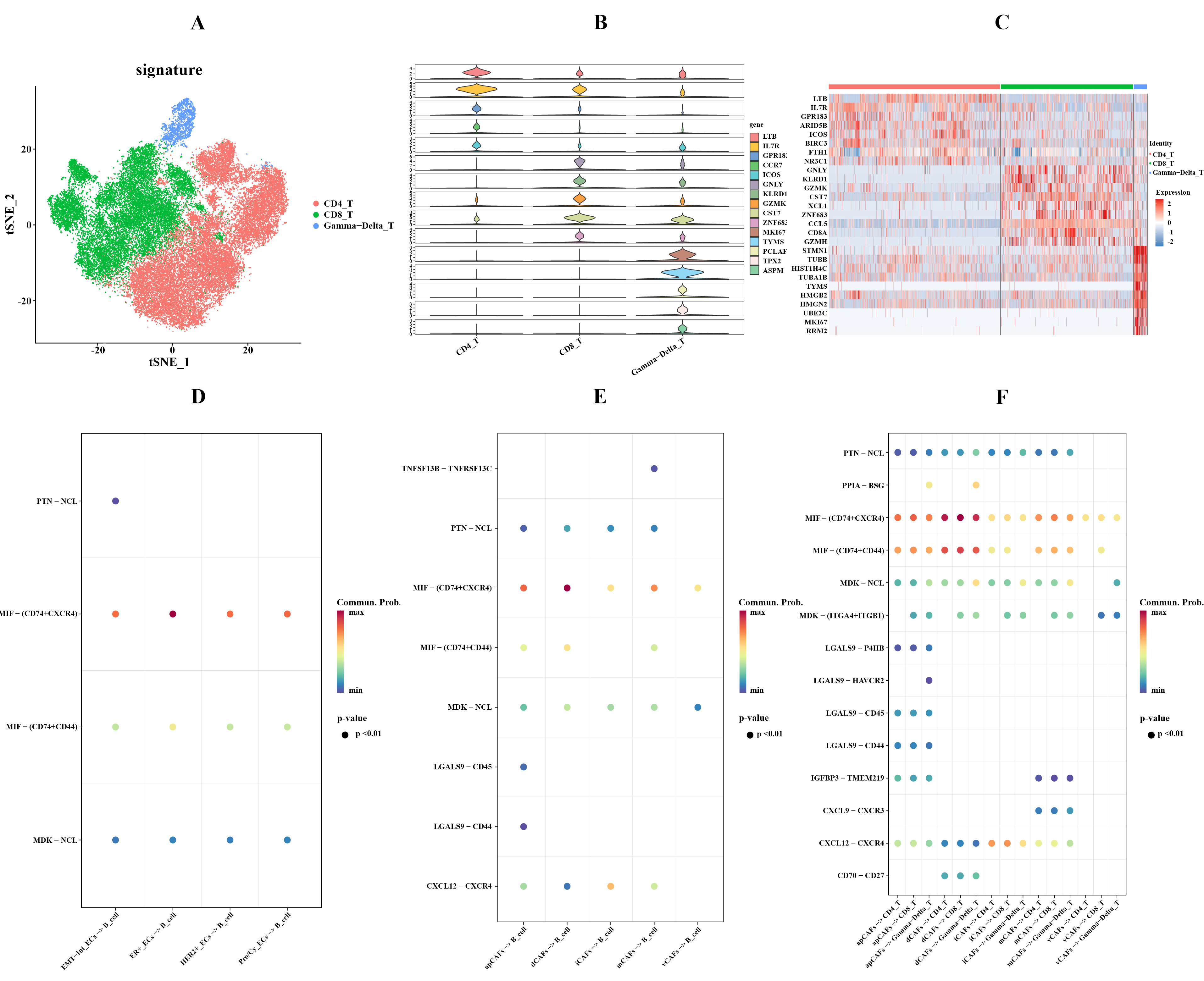

Supplement: Supplementary file 10 — Figure S10: Analysis of T‐cell subsets and their ligand‐receptor mediated interactions with epithelial cells and fibroblasts across BC subtypes. (A) t‐SNE plot displays the distribution of T cell subpopulations, labeled as CD4_T, CD8_T, Gamma‐Delta_T. (B) Violin plot shows the marker genes for T cell subpopulations. (C) Heatmap displays expression patterns of differentially expressed genes across subpopulations. (D) Diagram of ligand‐receptor mediated epithelial‐B cell interactions in HER2+BC. (E) Diagram of ligand‐receptor mediated CAF‐B cell interactions in TNBC. (F) Diagram of ligand‐receptor mediated CAF‐T cell interactions in TNBC. [file CAM4-15-e71600-s007.png]

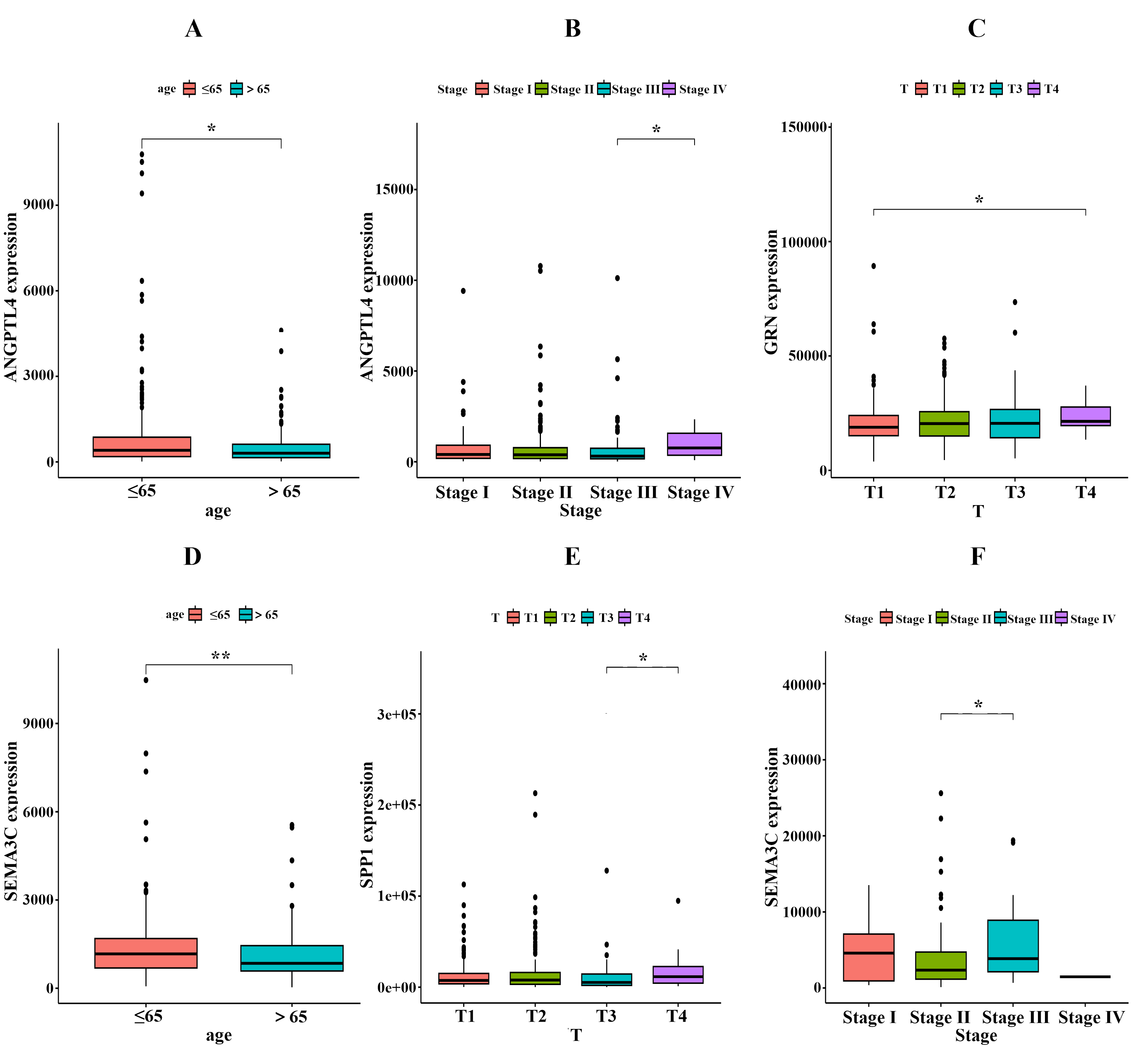

Supplement: Supplementary file 11 — Figure S11: Clinical relevance of key genes from CellChat‐derived ligand‐receptor pairs. (A) ANGPTL4 expression by age in ER+BC. (B) ANGPTL4 expression by pathological stage in ER+BC. (C) GRN expression by T‐stage. (D) SEMA3C expression by age in ER+BC. (E) SPP1 expression by T‐stage in ER+BC. (F) SEMA3C expression by pathological stage in TNBC. [file CAM4-15-e71600-s009.png]
